# Same Invasion, Different Routes: Helminth Assemblages May Favor the Invasion Success of the House Mouse in Senegal

**DOI:** 10.3389/fvets.2021.740617

**Published:** 2021-10-26

**Authors:** Christophe Diagne, Laurent Granjon, Caroline Tatard, Alexis Ribas, Arame Ndiaye, Mamadou Kane, Youssoupha Niang, Carine Brouat

**Affiliations:** ^1^CBGP, IRD, CIRAD, INRAE, Montpellier SupAgro, Univ. Montpellier, Montferrier-sur-Lez, France; ^2^Université Paris-Saclay, CNRS, AgroParisTech, Ecologie Systématique Evolution, Orsay, France; ^3^Parasitology Section, Department of Biology, Health Care and Environment, Faculty of Pharmacy and Food Science, Institut de Recerca de la Biodiversitat (IRBio), University of Barcelona, Barcelona, Spain; ^4^BIOPASS, CBGP-IRD, ISRA, UCAD, CIRAD, Campus de Bel-Air, Dakar, Senegal

**Keywords:** biological invasions, enemy release, gastrointestinal helminths, *Mastomys erythroleucus*, *Mus musculus domesticus*, spatial survey, spill-back

## Abstract

Previous field-based studies have evidenced patterns in gastrointestinal helminth (GIH) assemblages of rodent communities that are consistent with “enemy release” and “spill-back” hypotheses, suggesting a role of parasites in the ongoing invasion success of the exotic house mouse (*Mus musculus domesticus*) in Senegal (West Africa). However, these findings came from a single invasion route, thus preventing to ascertain that they did not result from stochastic and/or selective processes that could differ across invasion pathways. In the present study, we investigated the distribution of rodent communities and their GIH assemblages in three distinct zones of Northern Senegal, which corresponded to independent house mouse invasion fronts. Our findings first showed an unexpectedly rapid spread of the house mouse, which reached even remote areas where native species would have been expected to dominate the rodent communities. They also strengthened previous insights suggesting a role of helminths in the invasion success of the house mouse, such as: (i) low infestation rates of invading mice by the exotic nematode *Aspiculuris tetraptera* at invasion fronts—except in a single zone where the establishment of the house mouse could be older than initially thought, which was consistent with the “enemy release” hypothesis; and (ii) higher infection rates by the local cestode *Mathevotaenia symmetrica* in native rodents with long co-existence history with invasive mice, bringing support to the “spill-back” hypothesis. Therefore, “enemy release” and “spill-back” mechanisms should be seriously considered when explaining the invasion success of the house mouse—provided further experimental works demonstrate that involved GIHs affect rodent fitness or exert selective pressures. Next steps should also include evolutionary, immunological, and behavioral perspectives to fully capture the complexity, causes and consequences of GIH variations along these invasion routes.

## Introduction

Parasitism has been considered a key factor explaining the successful range expansion of many introduced species (1–3). Indeed, parasites may indirectly shape interactions among native and invasive host species—to the benefit of the latter—through different mechanisms. First, the “enemy release” hypothesis states that newly introduced host populations may benefit from an impoverishment or reduced infection levels of their original parasite communities; this reduction of their parasite burden may enhance host fitness and performance in the new environment, ultimately facilitating settlement and spread (4–7). Second, the “spill-over” hypothesis states that exotic hosts may introduce some of their coevolved parasites that may have negative impacts on native hosts in the introduction area (8–10). Finally, disease facilitation hypotheses state that introduced species may increase infection levels within native hosts, by either acting as additional competent reservoirs or vectors of local parasites [“spill-back” hypothesis; (11–13)], improving parasite circulation through habitat alteration (14), and/or impacting native host condition through stress induction (15).

The aforementioned hypotheses have received ever-increasing supports from studies comparing invasive populations across one of their expansion routes [e.g., (16–18)]. While this design requires substantial knowledge of the invasion history (19), it has the advantage of reflecting a spatial and temporal continuum in the invasion process (20)—which is fundamental to unravel the actual role of parasites in the successful spread of invaders. However, successful introduction and establishment of invading species may involve stochastic processes related to population bottlenecks (21, 22). Hence, contrasted population dynamics and parasitic pressure may differ between invasion routes of a given species, as evidenced in previous studies on the Australian cane toad (23, 24). Therefore, comparisons between invasion routes taken by an invasive species appear essential to ensure consistent supports, and then disentangle the consequences of stochastic and selective processes across invasion pathways. Yet, such comparative studies—particularly in the same expansion range—are still scarce as far as we are aware [but see (25)].

The house mouse (*Mus musculus domesticus*) is a worldwide commensal rodent that made use of human migrations to expand its distribution range (26). In Senegal, this species was introduced during the colonial period in coastal cities, and firstly in the region of Saint-Louis. From there, it has taken advantage of the improvement of transport infrastructures and human activities to spread along coastal areas to Dakar then eastwards at the beginning of the twentieth century (27). This expansion has resulted in the extirpation of native rodents (mostly *Mastomys erythroleucus*) from cities and villages at the invasion front (28). Previous studies investigated gastrointestinal helminth (GIH) assemblages of rodents along a well-defined invasion route of the house mouse in the Northern part of this country (29, 30). Patterns were consistent with the predictions of the “enemy release” hypothesis—with infection levels of GIH in invading mice remaining low at the invasion front compared with anciently invaded populations from the western coastal areas. While no support was found for the “spill-over” hypothesis, increased infection over time by the single shared GIH (a local cestode—*Mathevotaenia symmetrica*) within coexisting populations of native and invasive rodents at the invasion front brought support to the “spill-back” hypothesis.

In this study, we evaluated whether GIH patterns remain consistent when considering areas with different invasion histories, strengthening supports for parasite-related hypotheses explaining the invasion success of the house mouse. For this purpose, we extended the spatial coverage of the field-based correlative approach mentioned above (29, 30). We investigated the distribution of both rodent communities and their GIH assemblages along three distinct zones of Northern Senegal, including sites of the invasion front previously studied as well as newly sampled sites that were presumed to have been more recently invaded by the house mouse. Indeed, unraveling the role of parasites on the ongoing spread of successful invaders requires in-depth knowledge of contemporary host progression. Population genetics approaches revealed that Northern Senegal was mostly invaded by house mice from the region of Saint-Louis, although some admixture with house mice originated from the region of Dakar has however been evidenced (27). We may consider these zones as distinct invasion fronts from the same originally introduced house mouse population. Specifically, we aimed at testing the following hypotheses. Regarding the rodent community, we expected that the house mouse presence and/or relative abundance are consistent with the situation of the three study zones, in terms of connectivity with known or presumed routes of ongoing spread eastwards by house mice in the country—i.e., (i) presence and even dominance in (already invaded) sites well connected to the main road networks, and (ii) absence or presence in coexistence with dominant native rodent populations in sites far from the main road networks. Regarding the GIH community, we expected (i) lower parasitism levels in house mice from the more recently invaded sites in accordance with the “enemy release” hypothesis; (ii) high infection levels of native parasites in *M. erythroleucus* populations co-existing with house mice at invasion fronts consistently with the “spill-back hypothesis;” and (iii) no evidence that native rodents coexisting with house mice were infected by exotic GIHs, given no support was found for the “spill-over” hypothesis in the previous studies (29, 30).

## Materials and Methods

### Sampling Design

**Figure 1:**
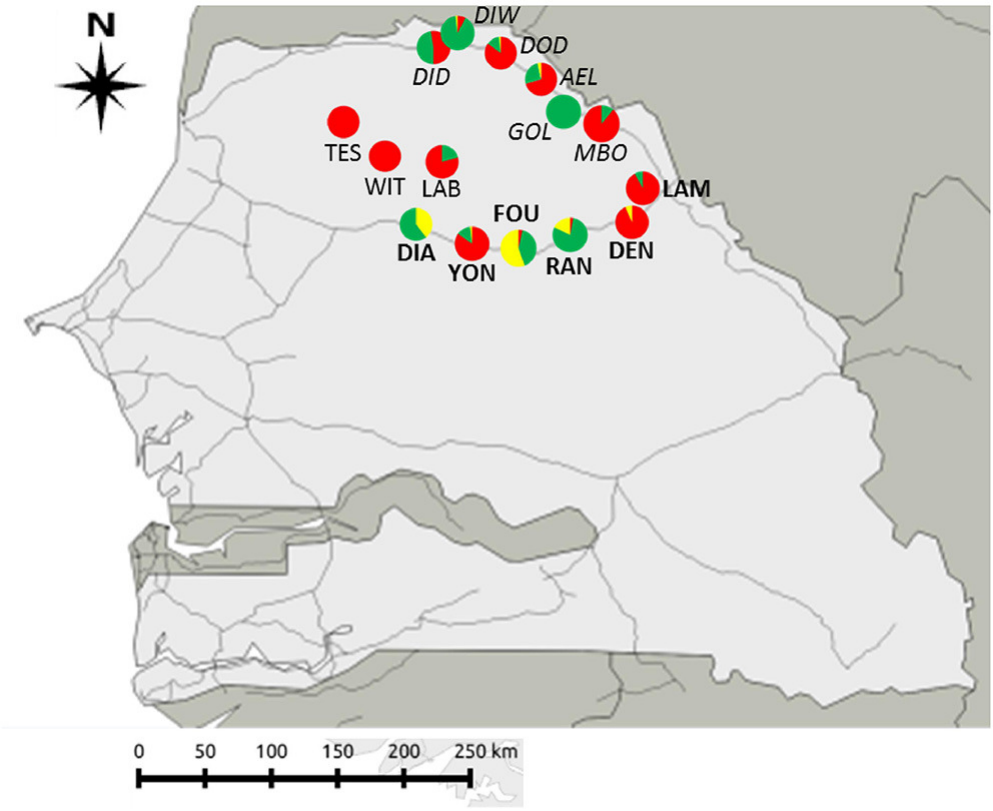
Geographic location of the 15 sites sampled across the three sampling zones in North Senegal. Rodent species: *Mus musculus domesticus* (in red); *Mastomys erythroleucus* (in green); *Arvicanthis niloticus* (in yellow). Zone “Central Ferlo” (sites in normal text): LAB, Labgar; TES, Tessekere; WIT, Widou Thiengoly. Zone “NR3” (sites in bold) belong to “NR3”: DEN, Dendoudi; DIA, Diagali; FOU, Fourdou; LAM, Lambango; RAN, Ranerou; YON, Yonofere. Zone “River valley” (sites in italics): AEL, Aere Lao; DOD, Dodel; DID, Diomandou Diery; DIW, Diomandou Walo; GOL, Gollere; MBO, Mboumba.

Sampled villages or towns (hereafter “sites”) were selected based on data from historical records, genetic analyses and ongoing longitudinal sampling surveys of rodent communities in Senegal carried out since the 1980's [CBGP - Small Mammal Collection, https://doi.org/10.15454/WWNUPO (27, 28)]. Some of the sites sampled (hereafter referred as “already studied sites”) had been previously surveyed for GIH assemblages in 2013 and 2016/2017, and were among those sites where signals of “enemy release” and “spill-back” were detected in coexisting native and invasive rodent populations (29, 30). In other sites (hereafter referred as “new sites”) where rodent communities may have been investigated in recent years, and GIH assemblages were not studied to date. We focused on three geographic areas (hereafter “sampling zones”) characterized by different levels of embeddedness into the network of modern roads, which imply different introduction opportunities and invasion histories for the house mouse (Figure 1):

(1) The first *sampling zone* (hereafter “River valley”) corresponds to six sites along the main National Road between Saint-Louis and Matam cities, in the Senegal River valley. In this zone, house mouse invasive populations have been well-established for <10 years and still co-exist with native *M. erythroleucus* populations. Among the six sampled sites, four were “already studied” for GIH assemblages (Aere Lao, Dodel, Diomandou Diery, and Diomandou Walo) and two were “new” sites (Gollere and Mboumba) where no or very few house mice were sampled in 2011 (28).
(2) The second *sampling zone* (hereafter “NR3”) corresponds to six sites along the eastern part of the National Road n°3 where surveys in 2011 indicated that some of them were not colonized by the house mouse at this time. This break in the house mouse distribution was explained by the late date (2011) at which the corresponding portion of the national road was asphalted (28). Among the six sites sampled, two were “already studied” sites for GIH assemblages (Dendoudi where house mice were sampled in 2013 and Lambango where no house mouse was found at the same time) and four were “new” sites (Diagali, Fourdou, Ranerou, and Yonofere) located along the road portion where the house mouse was not found in 2011.
(3) The third *sampling zone* (hereafter “Central Ferlo”) corresponds to three “new” sites (Labgar, Tessekere, and Widou Thiengoly) from the Central part of the Ferlo region, which is a pastoral zone isolated from the asphalted road networks to which it is connected only by few rural pathways on which commercial traffic has increased regularly over the last years (31). This area was never sampled for commensal rodents, but its isolation from the main road networks led us to expect that colonization by the house mouse—if any—would be recent, and then that coexisting native and invasive rodents might be found there.

We expected different situations regarding the respective distributions of the house mouse vs. native *M. erythroleucus* within “new sites:” (i) the house mouse is not established and native rodents are largely dominant or exclusively present, which would enable to confirm that GIH assemblages in native rodents differ from sites where native and invasive rodents co-exist; (ii) the house mouse co-exists with native rodent populations, and has founded large and dominant populations, which would enable to evaluate whether GIH assemblages reflect “enemy release” and “spill-back” patterns in other sites than those “already studied;” (iii) the house mouse is exclusively present, which would suggest a more rapid invasion than thought in the sites studied, given that they are located in remote areas or areas where the house mouse was not detected until a few years ago (28).

### Rodent Trapping, Sample Collection and GIH Identification

Trapping sessions were conducted inside human dwellings (e.g., houses, storehouses, or shops) between November 2016 and February 2017. A variable number of rooms were sampled (median ~ 60–80) at each site during sessions of one to three consecutive nights. We applied a standardized protocol [see (28, 29) for full details] using locally made wire-mesh live traps (8.5 × 8.5 × 26.5 cm) and Sherman folding box traps (8 × 9 × 23 cm) baited with peanut butter and fresh onions. Within each site, between 96 and 132 live-capture traps were set each afternoon, checked for night captures the following morning and then re-baited if necessary, with the aim to capture at least 20 adult rodents per species. Once trapped, rodent identification was performed based on morphology in the field (e.g., head-body, tail, hind foot, and ear lengths) and further molecular diagnosis in the lab if necessary [cytochrome b gene-based RFLP for species characterization in the genus *Mastomys* following (32)]. They were euthanatized by cervical dislocation and then weighed to the nearest 0.5 g, sexed, measured and dissected. None of the rodent species investigated here is officially protected, and every animal-related procedure was performed according to official ethical guidelines (33).

The digestive tract of each rodent was removed, unrolled and immediately stored in plastic universal vials containing 95% ethanol. In the lab, GIHs were carefully removed from the digestive tract of each rodent, then counted (except for helminths recovered from the stomach wall that were not quantifiable due to their physical alteration during helminth collection) and classified by morphotype. They were stored in 95% ethanol for further accurate identification as previously described [(29) and references therein].

Trapping campaigns within villages and private dwellings were conducted with prior explicit oral agreements from each relevant local authority (head of village) and individual householder, which were obtained after a comprehensive presentation of the project and its objectives. All protocols presented here were conducted following official regulations (Centre de Biologie pour la Gestion des Populations (CBGP): Agrément pour l'utilisation d'animaux à des fins scientifiques D-34-169-003) of the relevant institutional committee (Regional Head of the Veterinary Service, Hérault, France). All transfer and conservation procedures were performed in accordance with current Senegalese and French legislation.

### Data Analyses

All analyses were performed using specific packages in the R environment (34) and the Quantitative Parasitology software version 3.0 (35).

#### Habitat Characterization

Because they may correlate with the house mouse invasion history and its associated parasitism (29, 36), commensal habitat characteristics were recorded during trapping sessions in each sampling site. We recorded for each sampled room the material used for construction (sand, adobe, cement, sheet metal, and fibers) for each part of the dwelling (floor, walls, and ceiling), as well as the type of room (bedroom, restroom, shop, storage room, kitchen, etc.; Supplementary Material 1). We carried out a Multiple Component Analysis (MCA) with sampled rooms as observations and commensal habitat characteristics as variables. We statistically tested whether habitat-related characteristics were structured according to the *sampling zone* (River valley, Central Ferlo, NR3) by carrying out Between/Within-groups Analysis (BWA) with Monte Carlo tests (999 permutations) on the MCA outputs, considering the *sampling zone* as a grouping factor. Hence, we evaluated whether the graphical structure observed with the MCA was statistically significant. These analyses were made with the ade4 package v1.7-10 (37).

#### Structure of Rodent Communities

At the interspecific level, we carried out Pearson's Chi-squared test with Yates' continuity correction to assess whether and how relative abundances differed between *M. m. domesticus* and *M. erythroleucus* populations at each sampled site. At the intraspecific level, we tested if the sex-ratio and body mass differed among the *sampling zones*. We also evaluated this sex-ratio for each rodent population. To this extent, we used Chi-squared test for the sex-ratio and Kruskal–Wallis rank sum test (KW test) for the body mass separately for each rodent species. The analyses were performed using the MASS package (34, 38).

#### Structure of GIH Assemblages

For each rodent population at each site, we determined prevalence (percentage of infected rodents) and mean abundance [number of parasite(s) per rodent individual, infected or not] for each GIH collected. Furthermore, we investigated how the GIH assemblages were structured at both inter- and intra-specific levels across sampling zones. We thus performed a Correspondence Analysis (CA) based on a restricted dataset including only (i) infected hosts from sampling zones with more than 10 rodents captured and (ii) GIH taxa showing prevalence higher than 5% in the global dataset. We considered a GIH presence/absence dataset to prevent bias related to the GIH taxon for which exact counting could not be ensured (i.e., *Gongylonema*-like taxon; see results). First, we evaluated whether GIH assemblages were structured according to the host species. Under the “spill-over” hypothesis, we expected to detect at least one exotic GIH (i.e., brought by the house mouse) shared by both invasive and native rodents only in co-existence sites. Second, we assessed the potential structuring effect of the *sampling zone*; to avoid confounding effects from different host species captured in same *sampling zones*, we considered a novel variable combining both “host species” and “sampling zone” (hereafter “*rodent-based sampling zone*”). This variable consisted of five categories: (i) house mouse populations from River valley; (ii) house mouse populations from NR3; (iii) house mouse populations from Ferlo; (iv) native rodent populations from River valley; (v) native rodent populations from NR3—note that native rodents from Ferlo were excluded because of very low sample size (see Results). The statistical significance of both grouping factors, i.e., “host species” and “rodent-based sampling zone,” was tested independently using similar BWA as mentioned above.

#### Relations Between GIH Assemblages and Invasion Success

We used Generalized Linear Mixed Models (GLMMs) to evaluate whether spatial variations in GIH assemblages within rodent species and across sampling zones were consistent with the “enemy release” and “spill-back” processes. Models were performed separately for native and invasive rodent species as different outcomes were expected regarding both “enemy release” or “spill-back” processes (see Supplementary Material 1 for the datasets). (i) Under the “enemy release” hypothesis, we expected lower or similar GIH infection levels (overall or specific prevalence, abundance, and/or richness) in *M. m. domesticus* populations from “new” sites (which mostly belong to “NR3” and “Central Ferlo” zones where invasion by the house mouse is presumed to be more recent) compared with those from “already studied” sites (which mostly belong to the “River valley” zone) where signals of “enemy release” have been shown (29, 30). (ii) Under the “spill-back” hypothesis, we expected to detect higher infection levels (prevalence, abundance, and/or richness) of local GIHs in *M. erythroleucus* populations co-existing with house mice compared with non-invaded *M. erythroleucus* populations.

We considered overall prevalence (presence/absence of GIHs, combining all taxa), infracommunity species richness (number of GIH taxa in a single rodent host), specific prevalence (presence/absence of a given GIH taxon) and specific abundance (number of individuals of a given GIH taxon within all rodent hosts) as response variables. We assumed a binomial distribution (quasibinomial in cases of overdispersion) for prevalence data and a Poisson distribution (negative binomial in cases of overdispersion) for abundance and richness data. For ensuring sufficient statistical power, specific prevalence and abundance of GIH taxon were examined only if the prevalence exceeded 10% in each dataset. Similarly, rodent populations with too low sample size were excluded (i.e., Fourdou and Ranerou for *M. m. domesticus* analyses; Lambango, Labgar, and Yonofere for *M. erythroleucus* analyses). Sampling site was nested within sampling zone as a random factor. Six predictors were included in the GLMMs: (i) *two* intrinsic host factors (*gender* and *body mass*) known to be important drivers of parasitic infections in rodents (39, 40); (ii) the habitat characteristics—through numerical coordinates of each human dwelling extracted from the two first CA axes (Supplementary Materials 1, 2)—as the local environment likely influences the life cycle of GIHs (29); (iii) the *sampling zone* (“Central Ferlo,” “River valley,” and “NR3”)—each of which represents a distinct invasion front of the house mouse; and (iv) the *presence level* of the competitor rodent species (i.e., *M. m. domesticus* for *M. erythroleucus* GLMMs; *M. erythroleucus* for *M. m. domesticus* GLMMs) at each sampling site—which may be considered as a proxy of the invasion status given the ongoing spread of the house mouse is associated with extirpation of native rodent populations (28). For this latter predictor, invasive rodent populations were considered *not yet established* if there were no or very few rodents (≤6) captured in the sampled site (as in Diagali, Fourdou, Gollere, Mboumba, Ranerou; Table 1) or *co-existing* otherwise; native rodent populations were considered *low* if there were no or very few individuals (≤6) captured in the sampled site (as in Dendoudi, Labgar, Tessekere, Widou Thiengoly, Yonofere; Table 1) or *co-existing* otherwise.

**Table 1:** Number of rodent captured and analyzed per rodent species.

**Sampling zones/sites**	***Mus musculus domesticus***			***Mastomys erythroleucus***			***Arvicanthis niloticus***		
	**F**	**M**	**T**	**F**	**M**	**T**	**F**	**M**	**T**
Zone “River valley”	78 (66)	44 (34)	122 (100)	101 (70)	105 (65)	206 (135)	2 (0)	2 (0)	4 (0)
**Aere Lao**	26 (21)	15 (11)	41 (32)	10 (9)	5 (4)	15 (13)	1 (0)	1 (0)	2 (0)
Diomandou Diery	14 (12)	7 (4)	21 (16)	10 (8)	9 (9)	19 (17)	0 (0)	0 (0)	0 (0)
*Diomandou Walo*	4 (4)	5 (5)	9 (9)	23 (16)	24 (17)	47 (33)	0 (0)	1 (0)	1 (0)
**Dodel**	32 (27)	13 (10)	45 (37)	5 (5)	2 (2)	7 (7)	1 (0)	0 (0)	1 (0)
*Gollere*	0 (0)	0 (0)	0 (0)	34 (21)	36 (16)	70 (37)	0 (0)	0 (0)	0 (0)
*Mboumba*	2 (2)	4 (4)	6 (6)	19 (11)	29 (17)	48 (28)	0 (0)	0 (0)	0 (0)
Zone ′Central Ferlo′	40 (33)	35 (24)	75 (57)	3 (0)	3 (0)	6 (0)	0 (0)	0 (0)	0 (0)
**Téssékéré**	8 (15)	14 (8)	22 (23)	0 (0)	0 (0)	0 (0)	0 (0)	0 (0)	0 (0)
**Widou Thiengoly**	17 (6)	10 (10)	27 (16)	0 (0)	0 (0)	0 (0)	0 (0)	0 (0)	0 (0)
**Labgar**	15 (12)	11 (6)	26 (18)	3 (0)	3 (0)	6 (0)	0 (0)	0 (0)	0 (0)
Zone ′National Road 3′	60 (56)	43 (37)	103 (93)	25 (21)	35 (32)	60 (53)	25 (0)	22 (0)	47 (0)
**Dendoudi**	30 (28)	13 (11)	43 (39)	0 (0)	0 (0)	0 (0)	3 (0)	0 (0)	3 (0)
*Diagali*	0 (0)	0 (0)	0 (0)	5 (3)	10 (9)	15 (12)	10 (0)	13 (0)	23 (0)
*Fourdou*	1 (0)	0 (0)	1 (0)	3 (2)	8 (8)	11 (10)	11 (0)	4 (0)	15 (0)
**Lambango**	19 (19)	16 (13)	35 (32)	0 (0)	3 (0)	3 (0)	0 (0)	0 (0)	0 (0)
*Ranérou*	0 (0)	1 (0)	1 (0)	14 (13)	13 (11)	27 (24)	1 (0)	5 (0)	6 (0)
**Yonoféré**	10 (8)	13 (12)	23 (20)	3 (0)	1 (0)	4 (0)	0 (0)	0 (0)	0 (0)
All sites	176 (155)	119 (95)	295 (250)	129 (91)	143 (97)	272 (188)	27 (0)	24 (0)	51 (0)

*F, Females; M, Males; T, Total. Numbers in brackets indicate the number of individual rodents analyzed in the generalized linear mixed models. Sampling sites were put either in bold (when M. m. domesticus was statistically the dominant species) or in italics when native M. erythroleucus was statistically the dominant species (Chi squared tests; p < 0.05; when no statistical test was possible because of too low sample size from one of both rodent species, we based our decision on the raw number of sampled individuals). Note that three mice (Dodel, Dendoudi, and Tessekere) and one M. erythroleucus individual (Diagali) could not be sexed and were therefore not included in the table*.

A model selection approach was performed using the Akaike information criterion with correction for samples of finite size (AICc) as a goodness-of-fit indicator. The starting models included all predictors. Models with a ΔAICc <2 with respect to the model with the lowest AICc were selected and the most parsimonious of these models (i.e., the model with the fewest number of predictors and the higher variance explained) was chosen. The significance of explanatory variables and their interactions was determined by deletion testing and log-likelihood ratio tests. For each final model, linear regression residuals were checked to graphically ensure that all assumptions regarding normality, independence and the homogeneity of variance were satisfied (Supplementary Material 3). We also ensured that (i) there was no significant spatial autocorrelation using Moran's I test and (ii) no overdispersion has occurred (Supplementary Material 3). When needed, *post-hoc* comparisons were performed with Wilcoxon tests using Holm's correction method for *p*-value adjustment (95% family-wise confidence level). All analyses were performed using DHARMa (41), MuMIn v1.43.6 (42), and lme4 v1.1-8 (43) R packages.

## Results

### Habitat Characterization

We found that only 5% of the total variance of habitat characteristics was explained by the *sampling zone* (BWA: Monte-Carlo test, *p* = 0.001), indicating that habitats were similar with respect to the variables recorded in each zone (Supplementary Material 2). The first two axes of our MCA concentrated 45.5% (axis 1: 24.57%; axis 2: 20.98%) and were therefore used as habitat coordinates in our GLMMs. However, neither the graphical observation of the MCA output nor the numeric contribution of each variable to axis construction allowed to adequately interpret both axes in regards with the habitat typology.

### Structure of Rodent Communities

We captured a total of 622 rodents across the 15 sampled sites (Figure 1, Table 1): 298 *M. m. domesticus*, 273 *M. erythroleucus*, and 51 *A. niloticus*—note that the latter species is not a strictly-commensal rodent and was therefore excluded from further analyses. *Mus m. domesticus* was captured in almost all sampled sites, confirming the continuous spatial spread of this invasive rodent in North Senegal (30). However, the house mouse was absent from Diagali (“NR3”) and Gollere (“River valley”), and present at very low levels at Mboumba (*n* = 6; “River valley”), Ranérou (*n* = 1; “NR3”), and Yonofere (*n* = 1; “NR3”). Surprisingly, the invasive house mouse largely dominated the rodent communities from “Central Ferlo,” where only six *M. erythroleucus* individuals were collected in a single site (Labgar). Moreover, both native and invasive rodent populations co-exist at similar abundance levels only at Diomandou Diery (“River valley”). Indeed, all other sampled sites were significantly dominated by one species or the other (KW tests; *p* < 0.005; Table 1; Supplementary Material 4). *Mastomys erythroleucus* was the dominant species at only six of the 15 sampled sites (Table 1; Supplementary Material 4), and absent from Tessekere (“Central Ferlo”), Widou Thiengoly (“Central Ferlo”), and Dendoudi (“River valley”).

Within both host species, we found no significant difference across the three *sampling zones* for the body mass (*M. m. domesticus*: Chi-squared = 4.4442; *p* = 0.1084; *M. erythroleucus*: Chi-square = 3.7768; *p* = 0.0519). Regarding the sex-ratio, only house mouse populations from “River valley” (Chi-square = 10.47; *p* = 0.00121) and Dendoudi (Chi-square = 6.7209; *p* = 0.0095) were biased toward female individuals; all other native and invasive rodent populations showed balanced sex-ratio across all *sampling zones* (Supplementary Material 5).

### Structure of GIH Assemblages

We recorded seven taxa of GIHs across the three *sampling zones* (Tables 2, 3): *Aspiculuris africana, A. tetraptera, Gongylonema* sp., *Mathevotaenia symmetrica, Pterygodermatites senegalensis, Pterygodermatites* sp., *Syphacia obvelata*. Almost all the GIH identified here were already identified in the previous studies (29, 30)—although nine of the 15 sampled sites were not yet previously investigated for GIH. The only new taxon identified was *Pterygodermatites* sp. in a single rodent captured at Gollere (“River valley”). In *M. m. domesticus*, we found four GIH species: *A. tetraptera* (overall prevalence = 14%), *M. symmetrica* (20.4%), *P. senegalensis* (0.8%), and *S. obvelata* (1.6%). In *M. erythroleucus*, we collected five GIH species: *A. africana* (13.9%), *Gongylonema* sp. (18%), *M. symmetrica* (32.9%), *P. senegalensis* (0.5%), and *Pterygodermatites* sp. (0.5%). *Mathevotaenia symmetrica*, which was already found previously to be shared by both native and invasive rodent species, was detected in all *sampling zones* except in *M. m. domesticus* populations from “Central Ferlo.” The nematode *P. senegalensis* was also recorded in both host species, but only anecdotally at two sites from the “River valley” zone (one *M. erythroleucus* at Gollere, one *M. m. domesticus* at Diomandou Walo).

**Table 2:** Prevalence (in percentage with 95% confidence intervals calculated with Sterne's exact method) and abundances (mean ± S.D.) of gastrointestinal helminth (GIH) taxa collected from each *Mus musculus domesticus* population.

**Sampling zone**	**Site (*N*)**	***Aspiculuris tetraptera***	***Mathevotaenia symmetrica***	***Pterygodermatites senegalensis***	***Syphacia obvelata***
River valley	Aere Lao (32)	12.5% [4.39–28.14] (0.63 ± 1.91)	43.8% [27.85–61.04] (0.91 ± 1.49)		
	Diomandou Diery (16)		12.5% [2.27–37.16] (0.31 ± 1.01)		
	Diomandou Walo (9)		11.1% [0.57–44.34] (0.67 ± 2)	11.1% [0.57–44.34] (0.11 ± 0.33)	
	Dodel (37)	2.7% [0.14–14.37] (0.03 ± 0.16)	29.7% [17.17–45.91] (0.43 ± 0.83)		8.1% [2.25–21.35] (0.32 ± 1.51)
Central Ferlo	Labgar (23)	17.4% [6.17–38.87] (4.87 ± 15.30)	13.0% [3.66–32.35] (0.87 ± 3.35)		
	Tessekere (16)	37.5% [17.78–62.83] (9.5 ± 18.6)	6.3% [0.3–30.5] (0.13 ± 0.5)	6.3% [0.3–30.54] (0.07 ± 0.25)	
	Widou Thiengoly (18)	44.4% [23.65–66.97] (5.22 ± 10.54)	22.2% [7.97–47.14] (0.39 ± 0.98)		
National Road 3	Dendoudi (39)		17.9% [8.60–33.16] (1.15 ± 3.38)		
	Fourdou (1)	100.0% [5.01–100] (18 ± 0.00)			
	Lambango (32)		15.6% [6.37–32.57] (0.25 ± 0.62)		
	Yonoféré (20)	55.0% [32.00–75.57] (3.65 ± 4.88)			

*N, number of adult rodents examined for GIH content*.

**Table 3:** Prevalence (in percentage with 95% confidence intervals calculated with Sterne's exact method) and abundances (mean ± S.D.) of gastrointestinal helminth (GIH) taxa collected from each *Mastomys erythroleucus* population.

**Sampling zone**	**Site (*N*)**	***Aspiculuris africana***	***Mathevotaenia symmetrica***	***Gongylonema-like***	***Pterygodermatites senegalensis***	***Pterygodermatites sp*.**
River valley	Aere Lao (13)	53.8% [26.05–77.60] (24.31 ± 35.96)	46.2% [22.40–73.95] (5.08 ± 9.71)	23.1% [6.61–51.96]		
	Diomandou Diery (17)		35.3% [16.64–59.37] (7.12 ± 19.79)	23.5% [8.47–48.87]		
	Diomandou Walo (33)		30.3% [16.12–48.46] (2.66 ± 7.09)	3.0% [0.16–16.11]		
	Dodel (7)	14.3% [0.74–55.42] (0.71 ± 1.89)	42.9% [12.88–77.46] (1.14 ± 2.19)			
	Gollere (37)	43.2% [28.18–59.54] (3.62 ± 7.20)	32.4% [18.49–48.64] (2.13 ± 4.91)	56.8% [40.46–71.82]	2.7% [0.14–14.37] (0.05 ± 0.33)	2.7% [0.14–14.37] (0.03 ± 0.16)
	Mboumba (28)	3.6% [0.19–17.48] (0.03 ± 0.19)	32.1% [17.49–51.81] (2.32 ± 5.14)	21.4% [9.77–40.91]		
Central Ferlo	Labgar (6)	16.7% [0.86–58.86] (23.83 ± 58.38)	66.7% [27.14–93.71] (3.5 ± 6.22)			
National Road 3	Ranérou (24)		54.2% [33.89–73.34] (2.42 ± 4.15)			
	Yonoféré (4)	25.0% [1.28–75.13] (0.25 ± 0.5)	25.0% [1.28–75.13] (4.5 ± 9)			

*N, number of adult rodents examined for GIH content. No abundance data was reported for Gongylonema-like taxon as the parasite individuals were difficult to quantify*.

**Figure 2:**
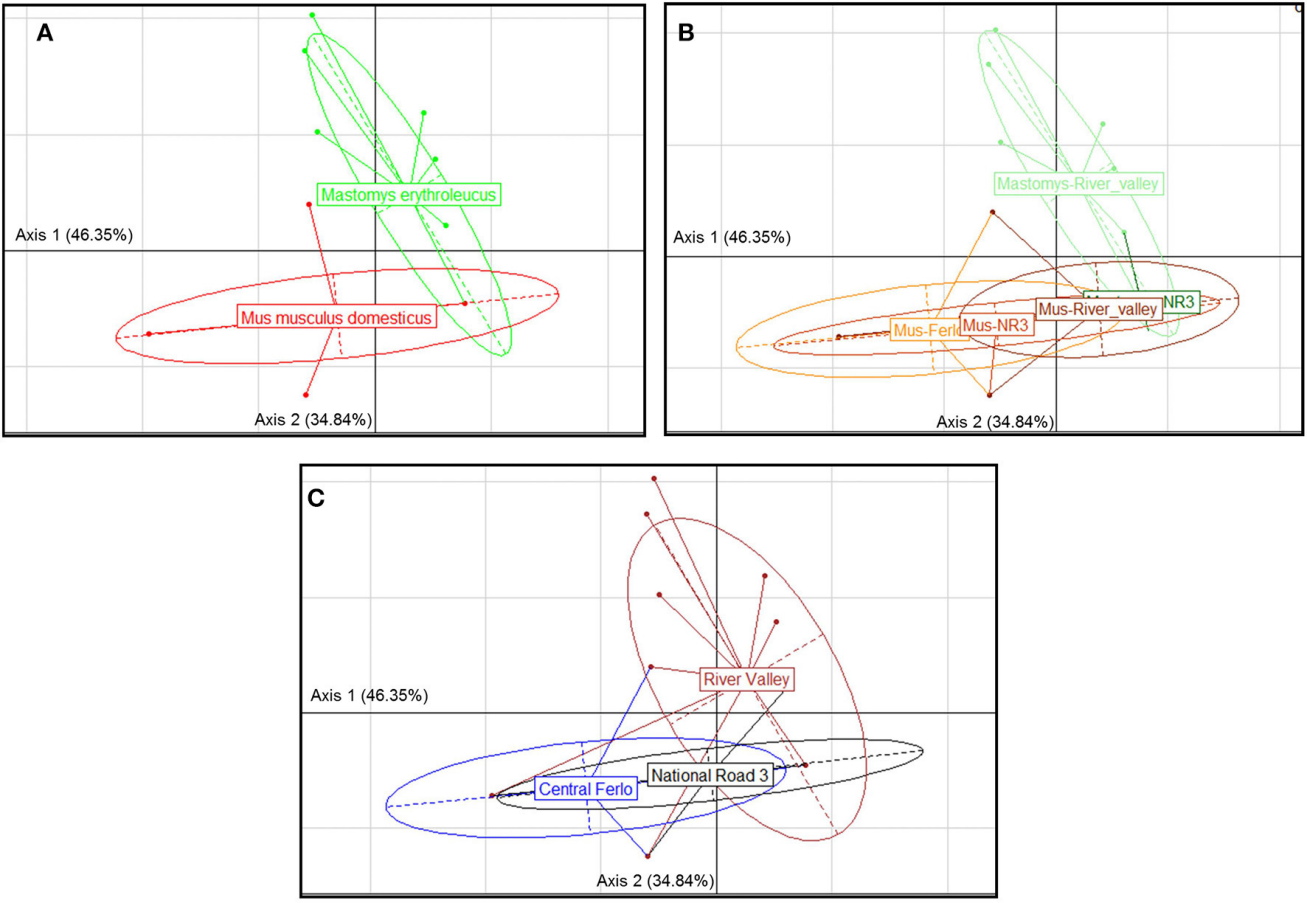
Correspondence analysis showing the structure of gastrointestinal helminth communities according (GIH) following **(A)** the rodent host species, **(B)** the species-specific category of sampling zones, and **(C)** the sampling zone. Between-within analysis showed significant structure for each factor grouping (Monte-Carlo test, *p*-value < 0.001). “Masto-NR3”: *Mastomys erythroleucus* populations from the zone *National Road 3*; “Masto-River_valley”: *M. erythroleucus* populations from the zone *River valley*; “Mus-Ferlo”: *Mus musculus domesticus* populations from the zone *Central Ferlo*; “Mus-NR3”: *M. m. domesticus* populations from the zone *National Road 3*; “Mus-NR3”: *M. m. domesticus* populations from the zone *River valley* “Masto-NR3” label is partly hidden by “Mus-River_valley” label.

**Figure 3:**
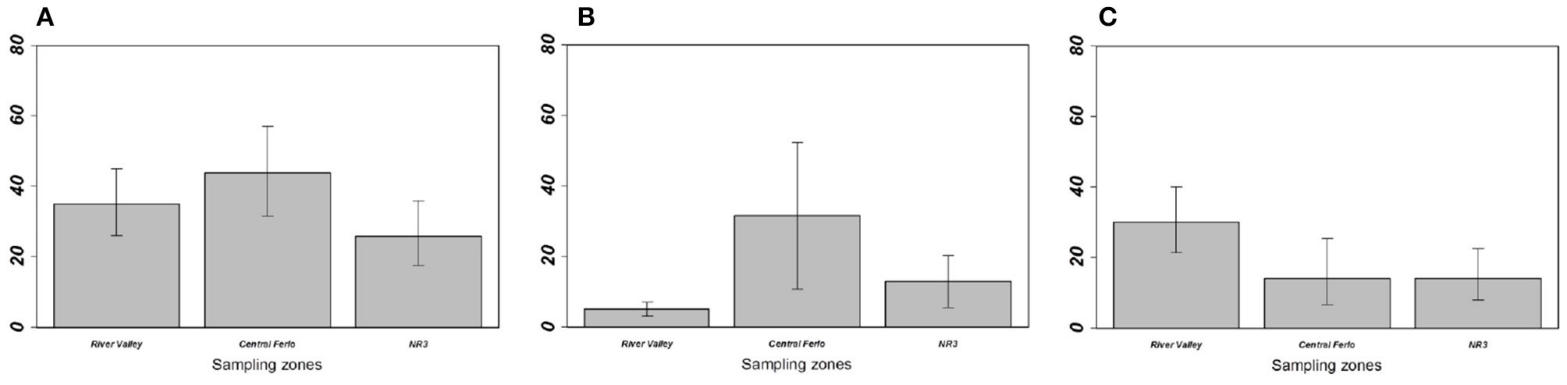
Prevalence of **(A)** overall gastrointestinal helminths, **(B)** *Aspiculuris tetraptera*, and **(C)** *Mathevotaenia symmetrica* between *Mus musculus domesticus* populations from the three sampling zones. Error bars represent 95% confidence intervals calculated with Sterne's exact method for prevalence data. NR3, National Road 3.

GIH assemblages strongly differed between host species (Figure 2A) with a single cestode (*M. symmetrica*) widely shared by both native and invasive rodent species. Comparison of GIH assemblages also revealed significant differences across *sampling zones* (Figure 2C), based on a combination of the relative host specificity of most GIHs recorded here as well as the spatial segregation observed in the rodent host distribution presented above (e.g., almost exclusive presence of *M. m. domesticus* in the “Central Ferlo”). At the intra-host level, GIH assemblages significantly differed according to the *rodent-based sampling zone* (Figure 2B). This was also explained by the fact that (i) the most prevalent nematode (*A. tetraptera*) was essentially recorded in “Central Ferlo” and the single shared cestode *M. symmetrica* was mostly detected at sites of “River valley” for *M. m. domesticus*; and that (ii) *A. africana* and *Gongylonema* sp. were almost exclusively recorded at sites of “River valley” for *M. erythroleucus* (Figure 3).

### Relations Between GIH Assemblages and Invasion Success

For *M. m. domesticus*, GLMMs revealed a significant effect of the *presence level* of *M. erythroleucus* on both prevalence (LRT = 5.823; *p* = 0.0106) and abundance (LRT = 6.532; *p* = 0.0155) of *A. tetraptera*, with lower parasitism rates in sites where house mice co-existed with native rodent populations (Table 4). This result was expected under the “enemy release” hypothesis, assuming that co-existence sites represent invasion fronts where the house mouse was recently established. Note that this *presence level* of *M. erythroleucus* was retained—with a marginally non-significant effect (LRT = 3.551; *p* = 0.0594)—in the most parsimonious model explaining variance in *M. symmetrica prevalence*, indicating a higher level of parasitism in house mice coexisting with native rodent populations. Host body mass was also correlated with both overall (LRT = 5.208; *p* = 0.0224) and *M. symmetrica* (LRT = 5.921; *p* = 0.0149) prevalence, with lower infection rates in lighter individuals (Table 4). We did not find any significant difference in infection levels between *sampling zones* in the most parsimonious model selected.

**Table 4:** Summary of the most parsimonious Generalized Linear Mixed Models (GLMMs) finally selected for both invasive *Mus musculus domesticus* and native *Mastomys erythroleucus*.

**Sss**	**Response variable**	**AICc (Δ)**	**R2**	**Predictors**	**LRT**	***p*-value**
*Mus musculus domesticus*	Overall prevalence	308.2 (0)	0.15	Body mass^b^	5.2087	0.0224
	*A. tetraptera* prevalence	158.4 (0)	0.56	Presence *M. erythroleucus*^a^	5.8231	0.0155
	A. tetraptera abundance	1,505.5 (0.46)	0.58	Presence *M. erythroleucus*^a^	6.532	0.0106
	*M. symmetrica* prevalence	249.2 (0)	0.11	Body mass^b^	5.9217	0.01496
				Presence *M. erythroleucus*^b^	3.5516	0.05949
*Mastomys erythroleucus*	Overall prevalence	220.4 (0)	0.35	Body mass^b^	8.1882	0.004216
				Zone (River valley > NR3)	5.3333	0.020922
				Host gender (Females>Males)	6.2654	0.012312
	Richness	362.2 (0.57)	0.31	Zone (River valley>NR3)	5.774	0.01626
	*M. symmetrica* prevalence	224.1 (0.58)	0.12	Body mass^b^	9.1844	0.00244
	*M. symmetrica* abundance	1,615.9 (0)	0.18	Body mass^b^	10.9607	0.000931
				Zone (River valley > NR3)	4.1054	0.042747
				Host gender (Females>Males)	4.4752	0.034391
				Habitat (axis 1)	1.4857	0.222888

*The sampling zone nested within study site was considered as a random factor. The information provided in brackets indicates the interpretation of the significance of the variable effect*.

*“>” indicates higher levels for the response variable*.

a, b *Refers to, respectively, negative or positive correlation between the response variable and the predictor*.

*AICc, Akaike's information criterion corrected for finite sample size; Δ, difference between the model selected and the model with the lowest AICc; LRT, Likelihood-ratio test*.

**Figure 4:**
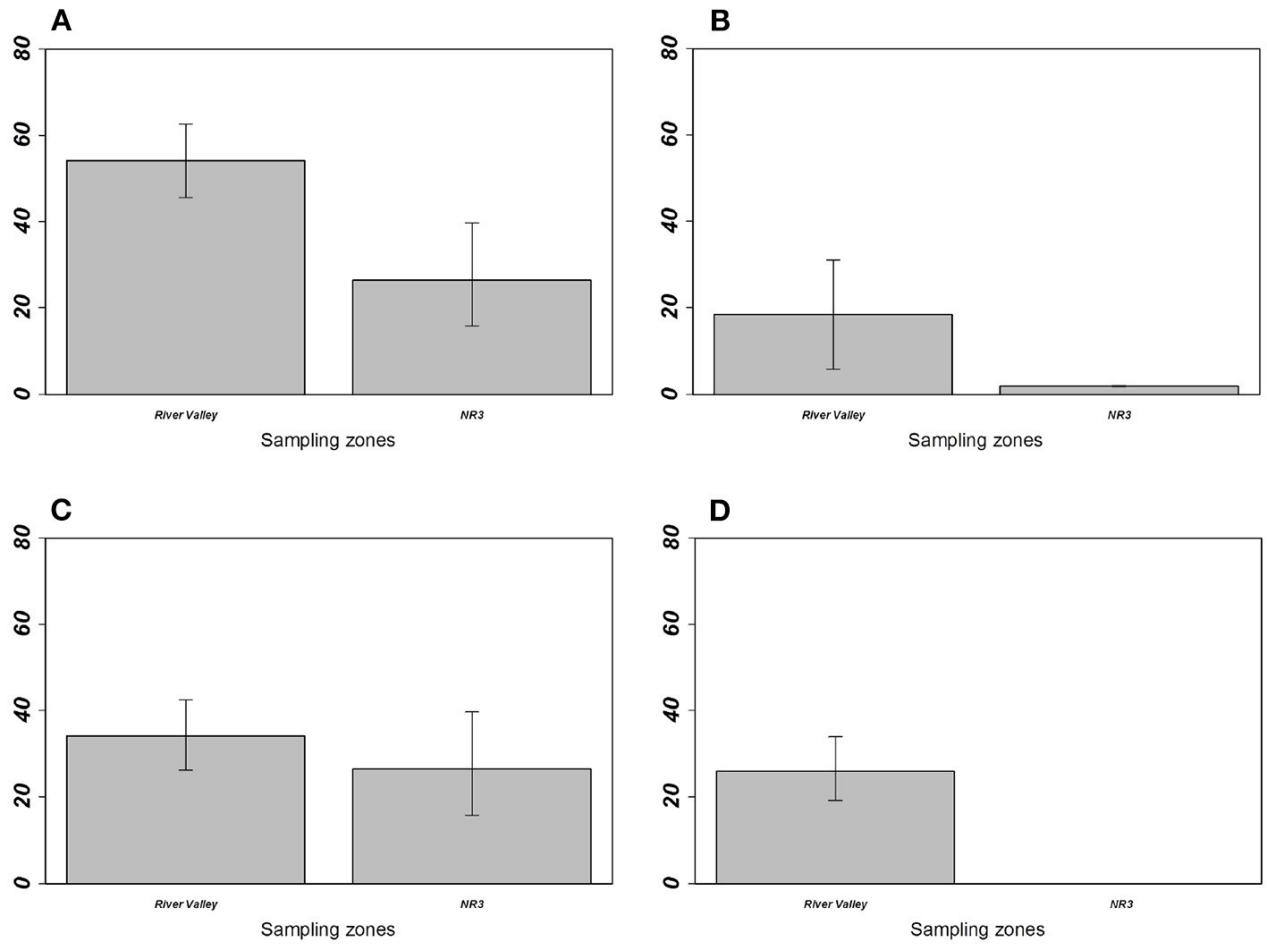
Prevalence of **(A)** overall gastrointestinal helminths, **(B)** *Aspiculuris africana*, **(C)** *Mathevotaenia symmetrica*, and **(D)** *Gongylonema*-like taxon between *Mastomys erythroleus* populations from River valley and National Road 3. Error bars represent 95% confidence intervals calculated with Sterne's exact method for prevalence data. *M. erythroleucus* population from Central Ferlo was not considered because of its low sample size (*n* = 6 individuals).

For *M. erythroleucus*, the most parsimonious models showed a significant effect of the *sampling zone* on overall prevalence (LRT = 5.333; *p* = 0.0209), individual richness (LRT = 5.774; *p* = 0.0162), and *M. symmetrica* abundance (LRT = 4.1054; *p* = 0.0427) with higher infection levels in “River valley” than in “NR3” (Table 4, Figure 4). Host gender also had a significant effect on overall prevalence (LRT = 6.265, *p* = 0.0123) and *M. symmetrica* abundance (LRT = 4.475; *p* = 0.0343), with higher infection levels in female rodents. Finally, we found that body mass was positively associated with higher overall prevalence (LRT = 8.188; *p* = 0.0042) as well as *M. symmetrica* prevalence (LRT = 9.184; *p* = 0.0024) and abundance (LRT = 10.960; *p* = 0.0009).

## Discussion

### Unexpectedly Rapid Spread of the House Mouse

Our results clearly showed that the invasion of the house mouse is still ongoing eastwards along various west-to-east axes within the northern part of the country. This finding confirms observations and expectations previously made (28, 30). As expected, the house mouse extended its invasion fronts in both Senegal River valley and NR3 zones, which are connected to the network of modern transport. Concomitantly, native *M. erythroleucus* populations experienced progressive extirpation from the local rodent communities. However, our sampling results also provided two surprising, yet highly interesting findings that challenged our initial expectations.

On the one side, while we thought that native rodent populations would have dominated the remote areas from Central Ferlo, we found that the house mouse was almost the only species present in these villages (only six native rodents caught in one of the three villages sampled). This result may testify for an intense and presumably rapid colonization of these villages by the house mouse, reflecting the ongoing process of opening up of this region. Indeed, Central Ferlo has experienced an intensification of its bottom-up urbanization and decentralization, accompanied by an increase of commercial exchanges (e.g., *via* the establishment of new village markets) and people's mobility over the last decade (31, 44). The similar habitat structure observed here between Central Ferlo and other sampling zones also supports this idea of a homogenization of development levels between these areas, in terms of human dwellings (Supplementary Material 2). The rapid establishment of the house mouse—along with the concomitant extirpation of native rodents—has been already observed at two sites from River valley (Aere Lao and Dodel) and Lambango (NR3) (30). The latter village can be considered as a village more connected to the road network than are the sites of the central Ferlo, but it is also one of the most distant eastwards on the NR3 axis. An alternative hypothesis may be that of an ancient establishment of the house mouse in the Central Ferlo, which could have occurred since decades thanks to traditional livestock and trade relationships with Northwestern cities long-colonized by the house mouse, such as Saint-Louis or Louga [*via* rural or urban markets of intermediate-sized localities like Dahra or Linguere; (31)].

On the other side, the house mouse was not (yet) detected at two sites located close to the national roads, i.e., Gollere (River valley, close to the second national road NR2) and Diagali (NR3). We identified two major, not mutually exclusive causes that may explain this pattern. First, the proximity of larger villages—such as Aere Lao or Mboumba in the River valley, or Yonofere in the NR3 zone—that concentrate most of the local trade and market activities may cause a reduction in the flows (of goods especially) in neighboring smaller villages. Second, local biotic characteristics—such as diversity and/or abundance of the invaded communities may influence the outcomes of invasions (45, 46). In this respect, the settlement of house mouse populations could have met biotic resistance from over-abundant and/or more complex small mammal communities in both sites. This hypothesis could be substantiated by the fact that (i) Gollere exhibited the highest trapping success of all the sampled sites (nearly 30%; http://projetcerise-ird-frb.fr–testifying for high abundance of native rodents there) and (ii) the relative abundance of house mouse populations was systematically lower in NR3 sites where both native *M. erythroleucus* and *A. niloticus* shared the same habitat—as seen at Diagali and Fourdou (Figure 1, Table 1).

From all the foregoing, a better understanding of the origins and mechanisms of the house mouse spread in Senegal would require a multidisciplinary approach combining rodent population genetics, behavioral ecology, social sciences and transportation geography. In any case, this spatial distribution of rodent species across the sampling zones offered us interesting opportunities to test the above-mentioned GIH-based hypotheses.

### Novel Supports for a Role of GIHs in the House Mouse Invasion Success

Our results confirm previous findings from the invasion route in North Senegal (29, 30). Indeed, we showed that heavier and female rodents were more frequently parasitized by GIHs, particularly regarding *M. symmetrica* (Table 4). The physiological, behavioral and ecological reasons that may explain these differences are thoroughly discussed elsewhere (36, 39, 47). More importantly, our findings provided new evidence that may support both “enemy release” and “spill-back” hypotheses along the spread of the house mouse in North Senegal.

On the one hand, house mouse populations from River valley and NR3 invasion fronts showed low GIH prevalence and abundance levels as expected in invasion fronts under the “enemy release” hypothesis (Figure 3, Table 1). This was particularly driven by *A. tetraptera*, which is the main GIH involved in the “enemy release” signal observed in spreading mouse populations over this area; the potential ways and implications of this parasite loss were already discussed (29, 30). Indeed, we previously found high prevalence of this exotic parasite on sites colonized long ago, while very low prevalence (similar to those found here in River valley and NR3 zones) were found on the invasion front. Surprisingly, *A. tetraptera* was found at much higher infection rates in sites from Central Ferlo. House mouse populations from this zone either did not experience any release of *A. tetraptera*, or have recovered high infection rates through ever-intense introduction events from source sites. Assuming that (i) release from this GIH is a marker of recent installation of the house mouse in the invaded areas and (ii) parasite recovery occurs in well-established mouse populations would provide support for a long-term establishment of the house mouse in the Central Ferlo [see (18, 48) for examples of parasite recovery over time]. In all cases, it seems that *A. tetraptera* infects invasive mice at high rates once native rodent populations are extirpated from the commensal habitat. Consistently, we found that the lower the presence level of native rodents, the higher the prevalence/abundance levels of *A. tetraptera* in house mouse populations. This concomitance between the absence of native rodents and the increase in *A. tetraptera* infection rates might well reflect a “parasite dilution” effect from native rodents (49, 50). Indeed, high infection rates of this GIH are exclusively observed in house mouse populations when native rodents are (almost) no longer present, suggesting that the release from *A. tetraptera* could be explained by native rodents acting as sink reservoirs. Elucidating whether “enemy release” actually occurs and which mechanisms (i.e., “dilution effect,” “parasite recovery over time”) are involved in the invasion success of the house mouse (if any) requires to combine (i) field surveys of ongoing “less-embedded” invasion fronts to assess the dynamics of this GIH over time and (ii) further experimental approaches to evaluate the effects of *A. tetraptera* on the house mouse fitness as well as the ability of this GIH to infect (or not) native rodents.

On the other hand, our analyses show that the abundance of *M. symmetrica*—the single GIH shared by both invasive and native rodents over all sampling zones—is higher in native rodent populations from River valley compared with those from NR3. A logical explanation could rely on environmental differences between both sampling zones. Nonetheless, we showed that the habitat structure did not significantly differ between sites from these zones (Supplementary Material 2)—although we are aware that the local environment was not fully captured with our habitat variables. Specific ecological characteristics may obviously contribute to this pattern. Scrutinizing our data reveals that *M. symmetrica* was not detected at NR3 sites (i.e., Diagali and Fourdou) dominated by the local rodent *A. niloticus* (Tables 1, 3). Both sites previously cited were also characterized by the anecdotal presence, or even absence of house mice. In addition, the circulation of this cestode which needs an intermediate insect host to complete its biological cycle (51). Therefore, both the distribution of intermediate hosts and rodent population structure may contribute to explain the observed low infestation opportunities for *M. erythroleucus* rodents. Alternatively, the higher abundance of *M. symmetrica* in native rodents from River valley compared with those from NR3 could bring new support to the “spill-back” hypothesis—given that the asphalted road network (and thus invasion opportunities by the house mouse) is more recent in the NR3 zone than in the River valley. In this context, infection opportunities of native rodents may have increased through direct (e.g., increase of infecting eggs in the local environment) and indirect (e.g., alteration of the behavior and/or body condition of local rodents through competition) mechanisms (3, 11, 12). *Mathevotaenia symmetrica* infection rates in native rodents were not statistically correlated with the presence level of the house mice probably because *M. erythroleucus* populations rapidly decreased as the house mouse is extending its distribution area (e.g., see the cases of Lambango and Central Ferlo discussed above). This rapid turnover in local rodent communities challenges the detection of any “spill-back” patterns—which needs the coexistence of both native and invasive rodent populations in sampling sites. Further surveys should prioritize those invasion front sites where the house mouse is not yet established (e.g., River valley: Gollere; NR3: Diagali and Fourdou), in order to decipher how *M. symmetrica* infection rates evolves in both native and invasive rodent populations as the invasion progresses. In addition, assessing a “spill-back” process driven by *M. symmetrica* in the invasion success of the house mouse would require experimentally evidencing the physiological or immunological effects of this cestode in native rodents.

## Conclusion

Our study strengthened findings and expectations made in previous studies focusing on the ongoing invasion success of the house mouse in North Senegal. First, the house mouse is still spreading eastwards, and has now unexpectedly colonized areas that were not considered as fully embedded to the market and road networks. The uneven rates of native-to-invasive species turnover observed across the sites sampled suggest that invasion dynamics and history of the house mouse may be markedly influenced at local scales by both human-mediated and ecological causes. Such processes deserve further exploration to understand and prevent further establishment of this invasive rodent. Second, our findings indicate that both “enemy release” and “spill-back” processes should be seriously considered when explaining the invasion success of the house mouse, provided we further examine the potential fitness consequences of parasite loss (in invasive rodents) or amplification (in native rodents). We are perfectly aware that we describe here only a part of the story in this complex rodent-GIH-invasion nexus. The study system considered here offers unique opportunities to examine more in depth the relationships between parasitism and invasion success. Next crucial steps should therefore also include evolutionary, immunological and behavioral perspectives in order to capture the complexity, causes and consequences of these interactive factors.

## Data Availability Statement

The datasets generated and analyzed in this study is available as a Supplementary Material 1. Information on available samples and collecting means for each rodent species are openly accessible at the ‘CBGP Database on Small Mammals’– http://bpm-cbgp.science/). All rodent and GIH sequences used in this study are available upon request and will be progressively deposited in public molecular databases.

## Ethics Statement

The animal study was reviewed and approved by Centre de Biologie pour la Gestion des Populations (CBGP): Agrément pour l'utilisation d'animaux à des fins scientifiques D-34-169-003 of the relevant institutional committee (Regional Head 191 of the Veterinary Service, Hérault, France). All transfer and conservation procedures were performed in accordance with current Senegalese and French legislation.

## Author Contributions

CB, CD, and LG designed the sampling strategy and managed the global project. CD, LG, MK, and YN managed the field sampling and data collection. CD carried out parasite collection, analyzed the data, and interpreted all results with AR, CB, and LG. AN, CD, and CT performed the molecular analyses. AR and CD performed morphological identification. CD, CB, and LG wrote the first draft of the manuscript with inputs from all the co-authors. All authors read and approved the final version of the manuscript.

## Funding

This work was supported by the CERISE project (funded by the Fond Francais pour l'Environnement Mondial *via* the Fondation pour la Recherche sur la Biodiversite': AAP-SCEN-20B III) and the Labex DRIIHM, French programme Investissements d'Avenir (ANR-11-LABX-0010) managed by the Agence Nationale pour la Recherche (ANR). The French Research Institute for Development (IRD) provided the funding for a postdoctoral position.

## Conflict of Interest

The authors declare that the research was conducted in the absence of any commercial or financial relationships that could be construed as a potential conflict of interest.

## Publisher's Note

All claims expressed in this article are solely those of the authors and do not necessarily represent those of their affiliated organizations, or those of the publisher, the editors and the reviewers. Any product that may be evaluated in this article, or claim that may be made by its manufacturer, is not guaranteed or endorsed by the publisher.

## Supplementary Material

Supplementary Material 1 Datasets considered for the Generalized Linear Mixed Models (GLMMs) and the Multiple Component Analysis (MCA). Datasets for GLMMs are provided specifically for *Mus musculus domesticus* and *Mastomys erythroleucus* as analyses were made separately for native and invasive rodent species.

Supplementary Material 2 Multiple Component Analysis (MCA) showing the difference in habitat structure across the three sampling zones: Central Ferlo (blue), NR3 (black), and River valley (red). Percentage in brackets represent the variance explained by each axis. We considered sampled rooms as observations and commensal habitat characteristics as variables (see Supplementary Material 1).

Supplementary Material 3 Validity checking of the most parsimonious Generalized Linear Mixed Models (GLMMs) finally selected for both invasive *Mus musculus domesticus* and native *Mastomys erythroleucus* datasets. The ratio between residual deviance and degrees of freedom was considered to evaluate the potential overdispersion. Moran's test was used to ensure that there was no significant spatial autocorrelation. We graphically checked the residuals to ensure their independency, heteroscedasticity, and normality.

Supplementary Material 4 Outputs from Chi-squared tests evaluating the difference between host sample sizes in each rodent population. Mus, *Mus musculus domesticus*; Mastomys, *Mastomys erythroleucus*; df, degrees of freedom; Ferlo, Central Ferlo; NR3, National Road 3; RV, River valley. Numbers in brackets indicate the sample size. Only sites for which statistical comparisons could be made (i.e., both rodent populations with sufficient number of captured individuals) between *M. m. domesticus* and *M. erythroleucus* populations are displayed. The other sites were mostly or exclusively dominated by one or the other rodent species (see Table 1).

Supplementary Material 5 Outputs from Chi-squared tests evaluating the sex-ratio for each rodent population. “balanced” indicates no statistical gender difference in the number of captured individuals within the rodent population; “biased (females)” indicates that the sex-ratio is biased toward female individuals. Results are significant when *p*-value < 0.05. df, degrees of freedom.
