# Supplementary material for: Same Invasion, Different Routes: Helminth Assemblages May Favor the Invasion Success of the House Mouse in Senegal
Source: Front Vet Sci. 2021 Oct 26;8:740617. doi: 10.3389/fvets.2021.740617 (PMC8576305; doi:10.3389/fvets.2021.740617)

Supplementary Material 2. Multiple Component Analysis (MCA) showing the difference in habitat structure across the three sampling zones: Central Ferlo (blue), NR3 (black) and River valley (red). Percentage in brackets represent the variance explained by each axis. We considered sampled rooms as observations and commensal habitat characteristics as variables (see Supplementary Material 1).

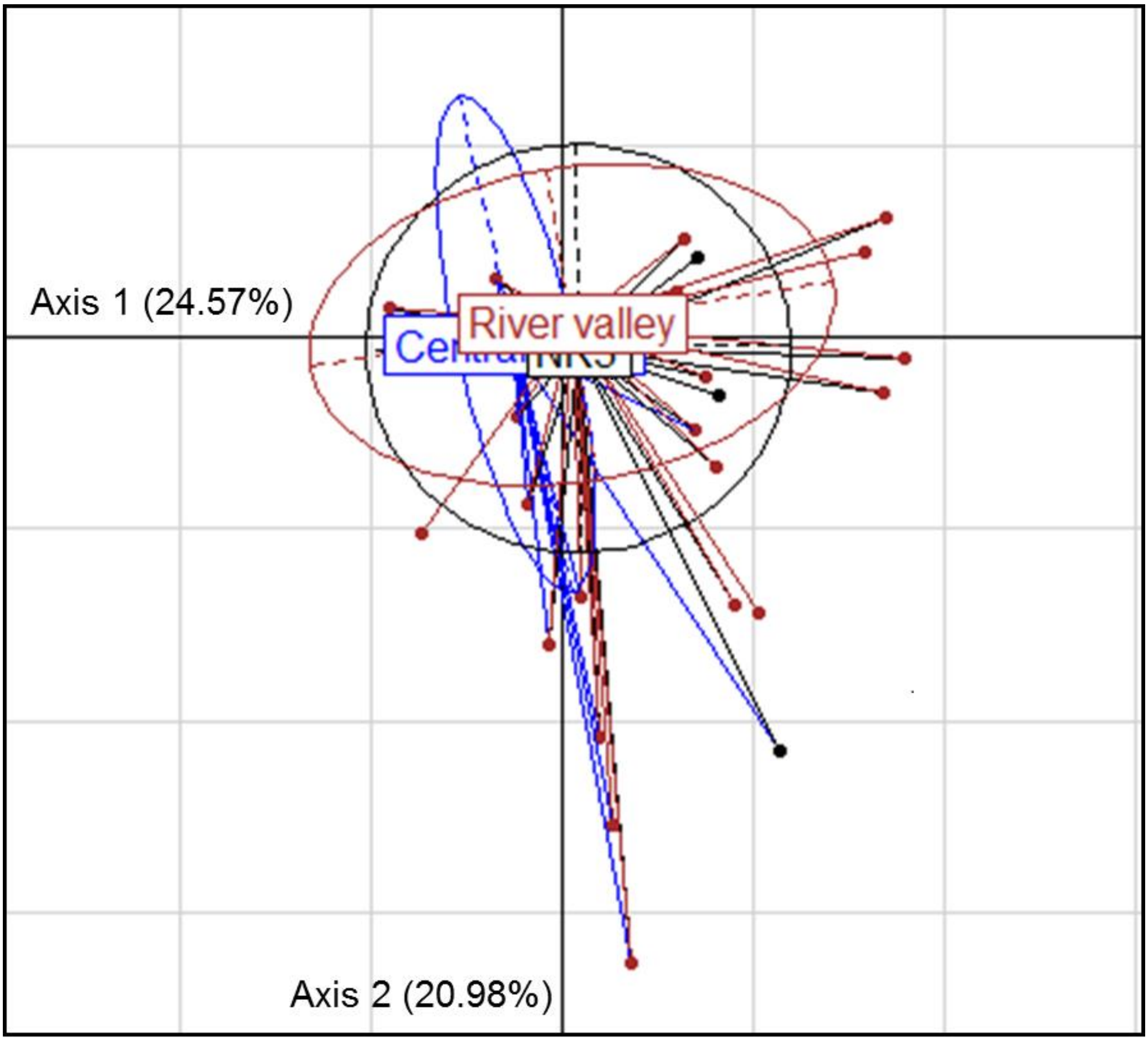

Supplement: Supplementary Material 2 — Multiple Component Analysis (MCA) showing the difference in habitat structure across the three sampling zones: Central Ferlo (blue), NR3 (black), and River valley (red). Percentage in brackets represent the variance explained by each axis. We considered sampled rooms as observations and commensal habitat characteristics as variables (see Supplementary Material 1). [file Data_Sheet_2.pdf]
