# Supplementary material for: Same Invasion, Different Routes: Helminth Assemblages May Favor the Invasion Success of the House Mouse in Senegal
Source: Front Vet Sci. 2021 Oct 26;8:740617. doi: 10.3389/fvets.2021.740617 (PMC8576305; doi:10.3389/fvets.2021.740617)

Supplementary Material 3. Validity checking of the most parsimonious Generalised Linear Mixed Models (GLMMs) finally selected for both invasive *Mus musculus domesticus* and native *Mastomys erythroleucus* datasets. The ratio between residual deviance and degrees of freedom was considered to evaluate the potential overdispersion. Moran's test was used to ensure that there was no significant spatial autocorrelation. We graphically checked the residuals to ensure their independency, heteroscedasticity and normality.

Part 1: Models selected for *Mus musculus domesticus* dataset

Model: Overall prevalence

- Residual deviance: 284.15 on 244 degrees of freedom (ratio: 1.17)
- Moran's I test for spatial autocorrelation:

observed = -0.0090740, expected = -0.0040486, sd = 0.0090662, *p-value = 0.5794*

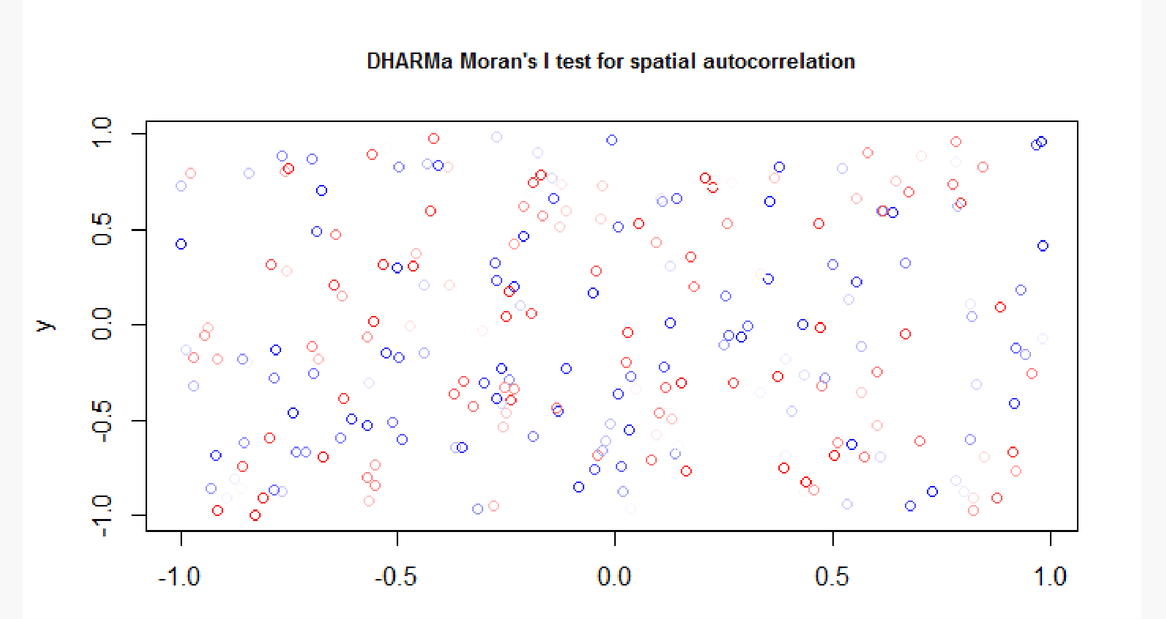

- Graphical checking of the residuals

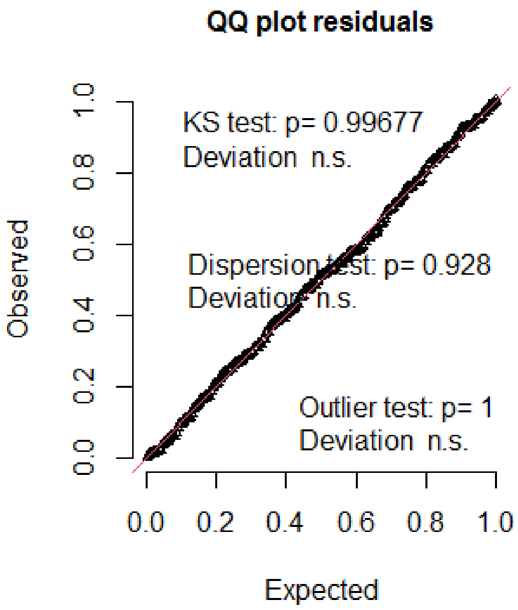

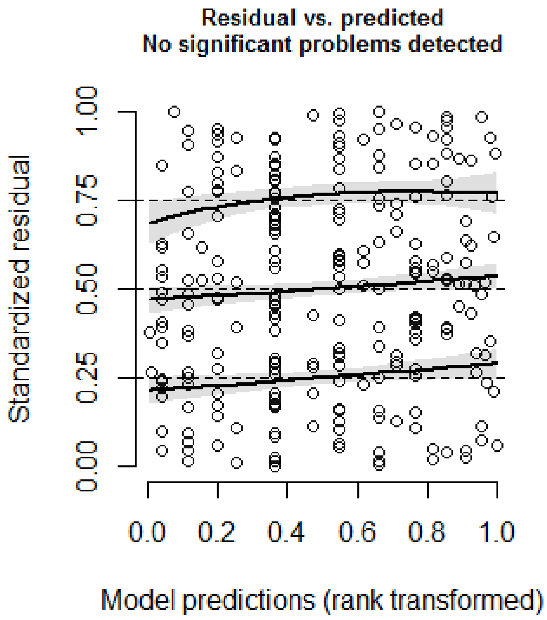

Model: *Aspiculuris tetraptera* prevalence

- Residual deviance: 132.079 on 244 degrees of freedom (ratio: 0.541)
- Moran's I test for spatial autocorrelation:

observed = -0.0115021, expected = -0.0040486, sd = 0.0085151, *p-value* = 0.3814

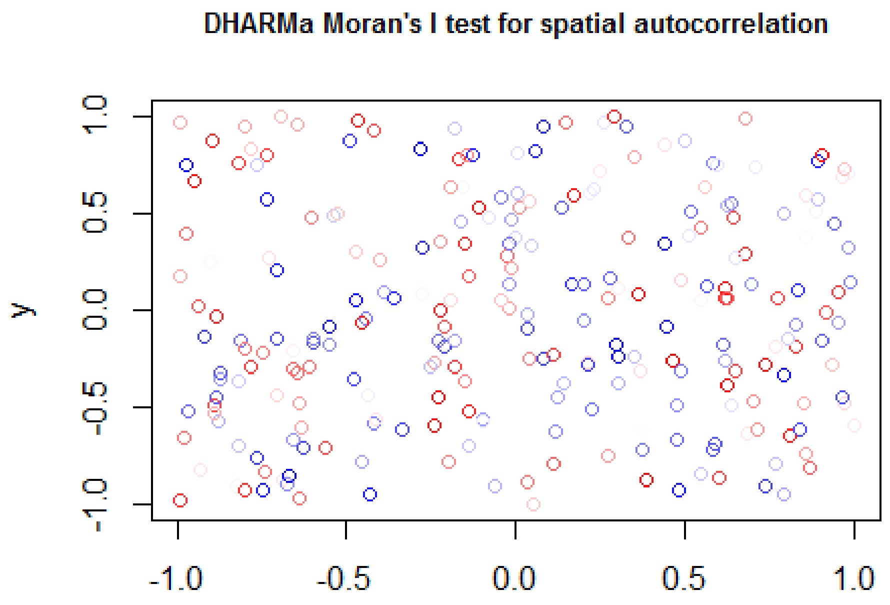

- Graphical checking of the residuals

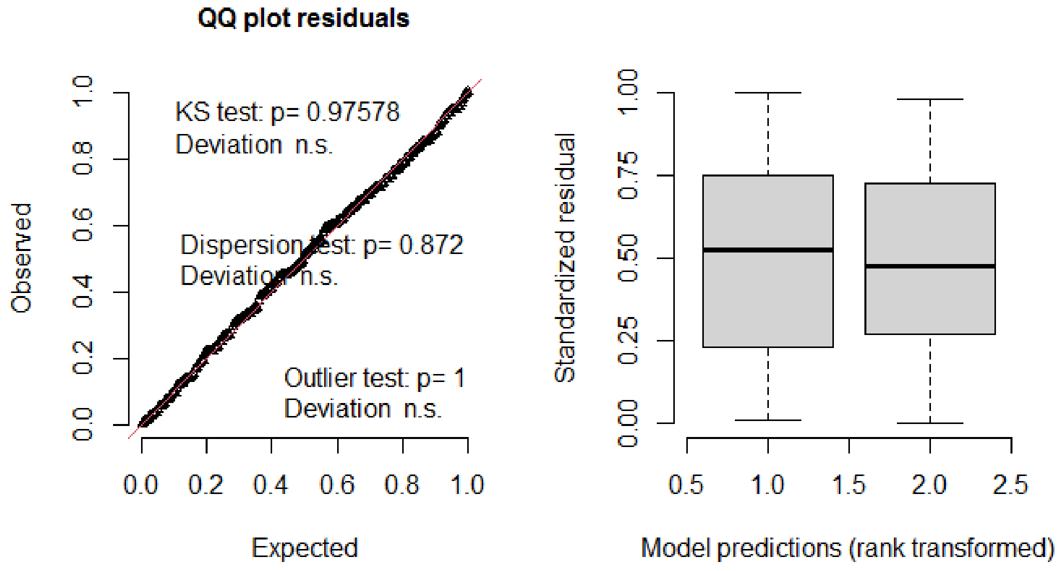

Model: *Aspiculuris tetraptera* abundance

- Residual deviance: 69.92 on 244 degrees of freedom (ratio: 0.287)
- Moran's I test for spatial autocorrelation:

observed = -0.0060397, expected = -0.0040486, sd = 0.0088094, *p-value = 0.8212*

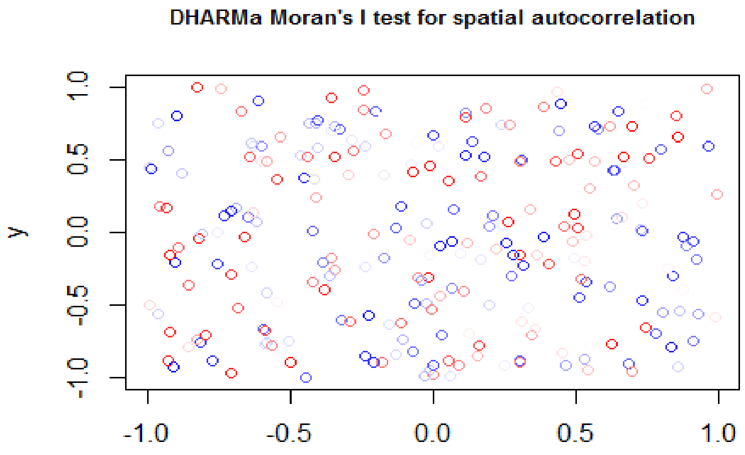

- Graphical checking of the residuals

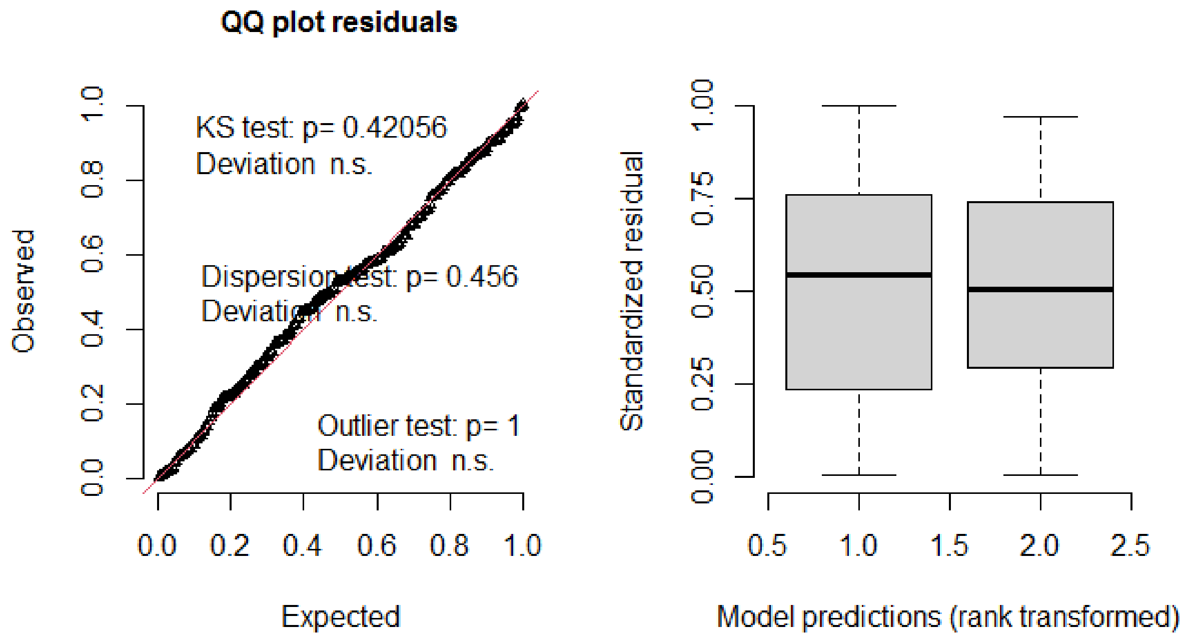

<u>Model</u>: *Mathevotaenia symmetrica* prevalence

- Residual deviance: 151.105 on 243 degrees of freedom (ratio: 0.622)
- Moran's I test for spatial autocorrelation:

observed = -0.0171457, expected = -0.0040486, sd = 0.0075300, *p-value = 0.08198*

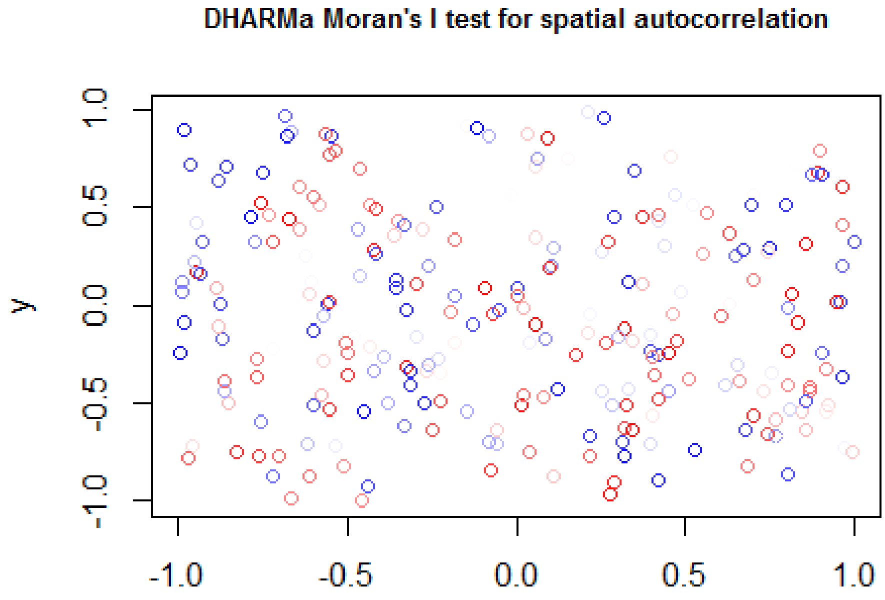

- Graphical checking of the residuals

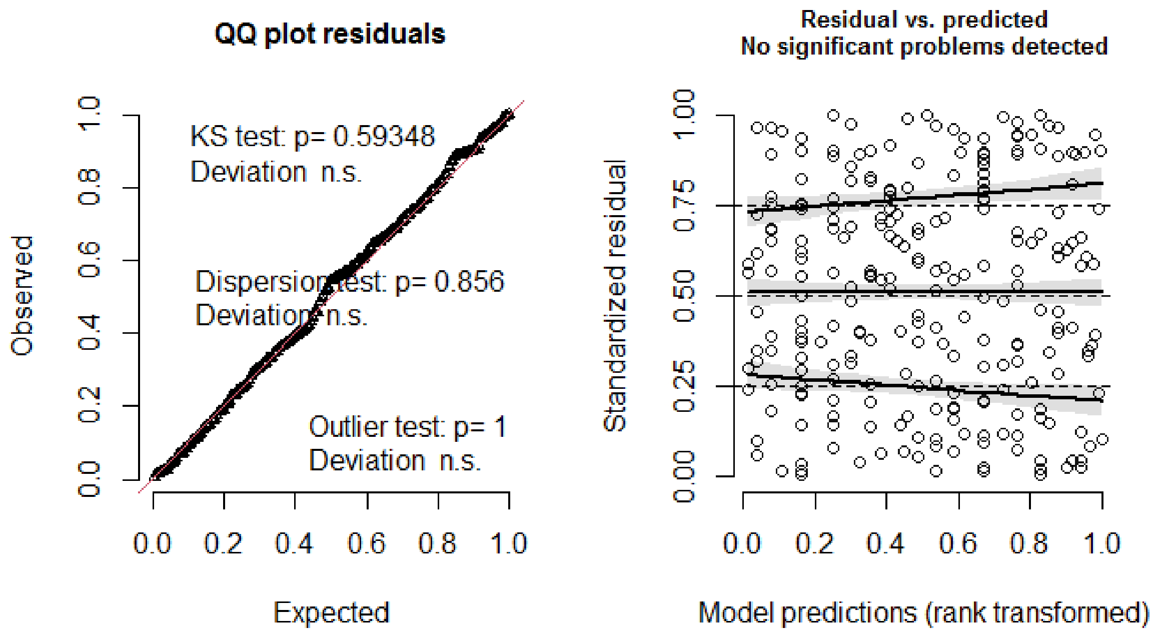

Mastomys erythroleucus

Model: Overall prevalence

- Residual deviance: 195.435 on 175 degrees of freedom (ratio: 1.117)
- Moran's I test for spatial autocorrelation:

observed = 0.0168458, expected = -0.0055556, sd = 0.0104662, *p-value = 0.03233*

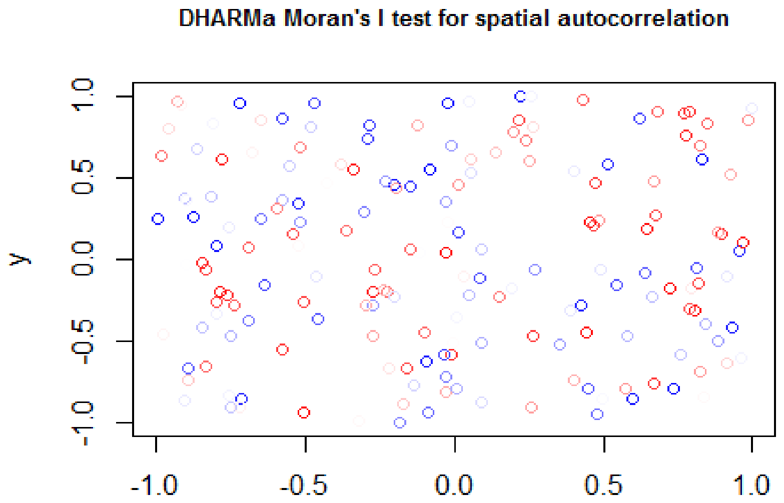

- Graphical checking of the residuals

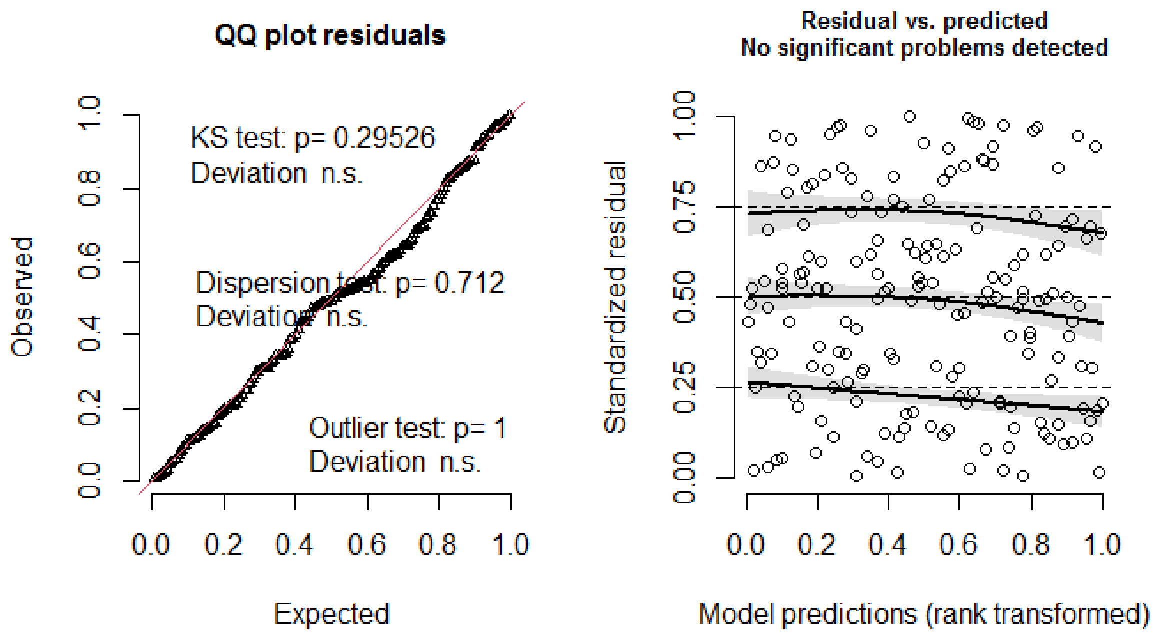

Model: Individual richness

- Residual deviance: 144.176 on 177 degrees of freedom (ratio: 0.815)
- Moran's I test for spatial autocorrelation:

observed = -0.0026373, expected = -0.0055556, sd = 0.0105807, *p-value = 0.7827*

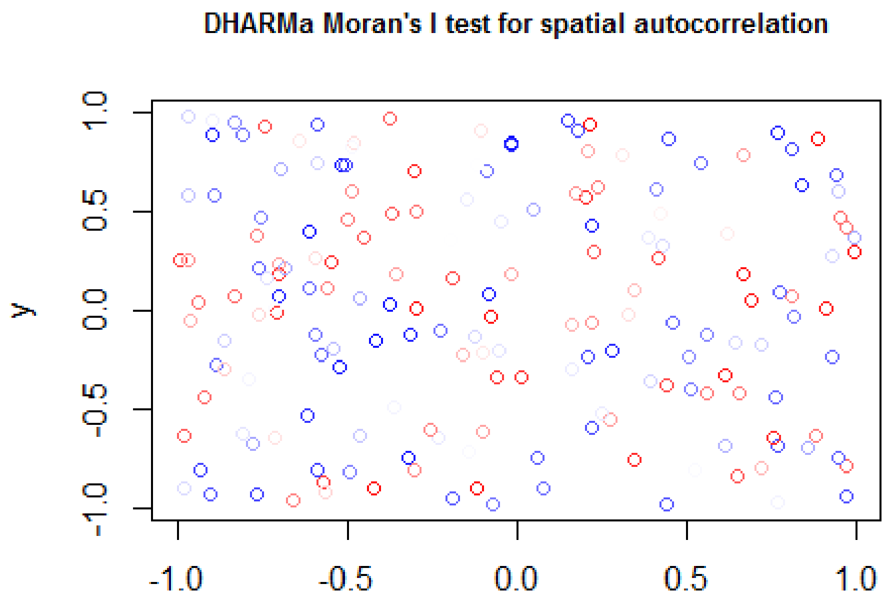

- Graphical checking of the residuals

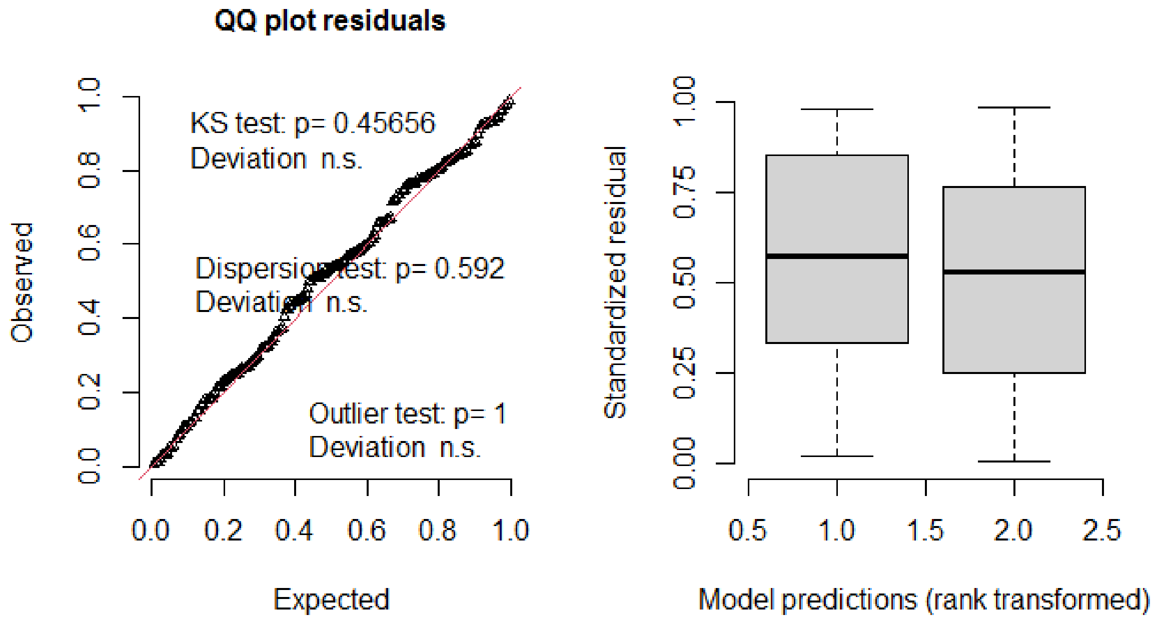

Model: *Mathevotaenia symmetrica* prevalence

- Residual deviance: 210.319 on 177 degrees of freedom (ratio: 1.188)
- Moran's I test for spatial autocorrelation:

observed = 0.00026427, expected = -0.00555556, sd = 0.01187797, *p-value = 0.6242*

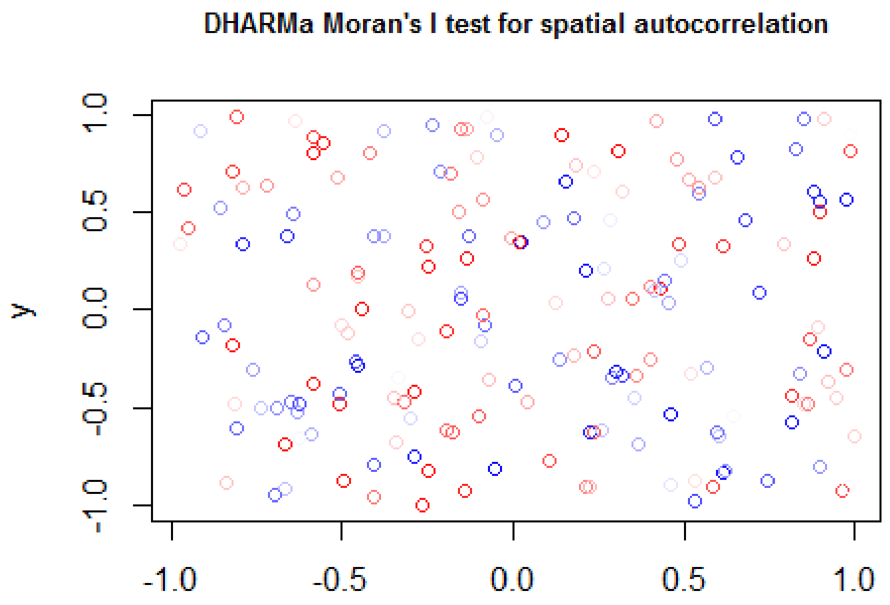

- Graphical checking of the residuals

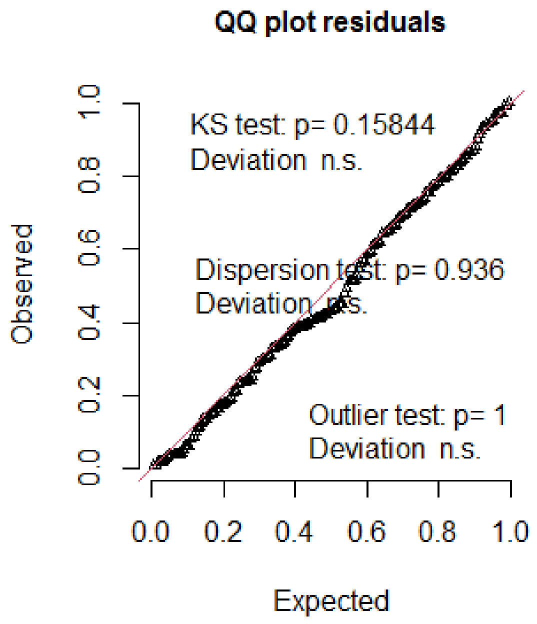

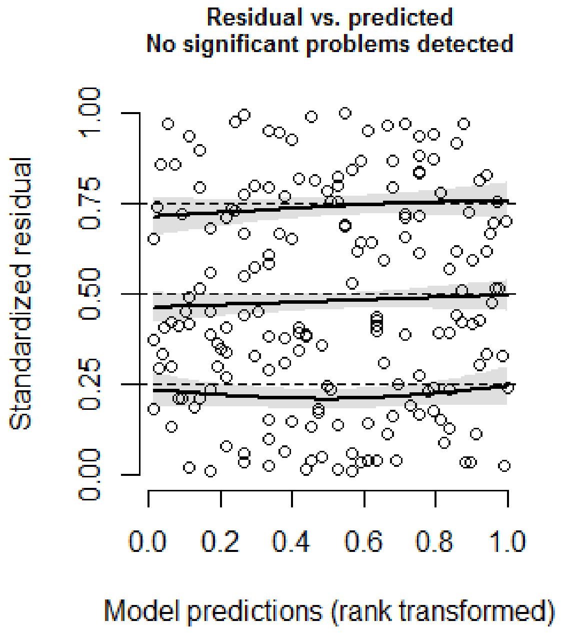

Model: *Mathevotaenia symmetrica* abundance

- Residual deviance: 210.319 on 177 degrees of freedom (ratio: 1.188)
- Moran's I test for spatial autocorrelation:

observed = 0.0055476, expected = -0.0055556, sd = 0.0110715, *p-value = 0.3159*

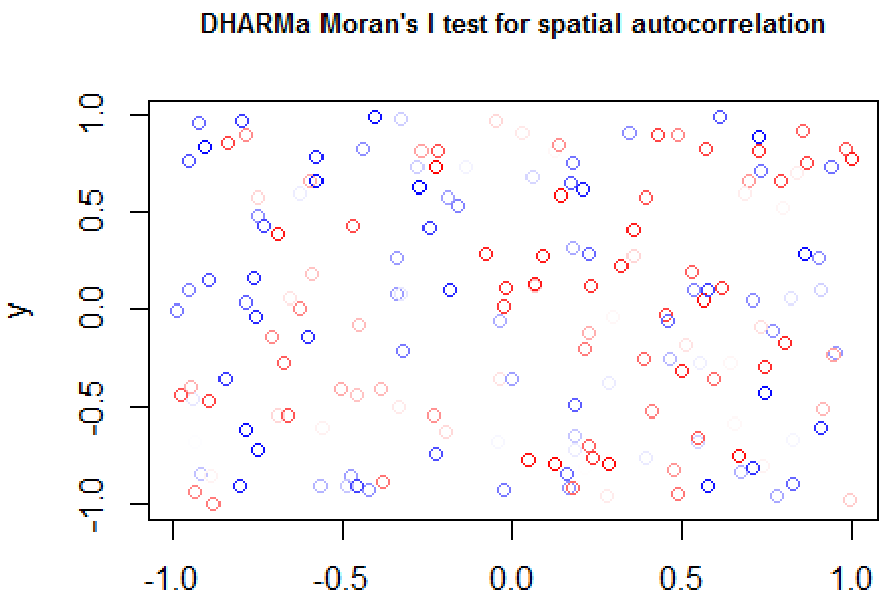

- Graphical checking of the residuals

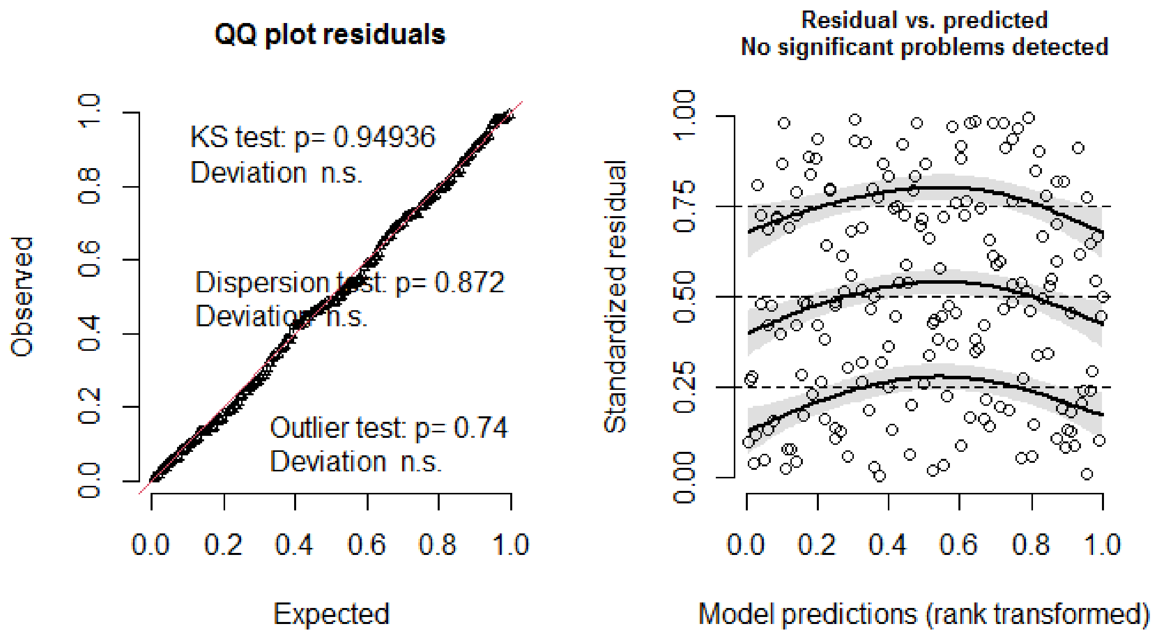

Supplement: Supplementary Material 3 — Validity checking of the most parsimonious Generalized Linear Mixed Models (GLMMs) finally selected for both invasive Mus musculus domesticus and native Mastomys erythroleucus datasets. The ratio between residual deviance and degrees of freedom was considered to evaluate the potential overdispersion. Moran's test was used to ensure that there was no significant spatial autocorrelation. We graphically checked the residuals to ensure their independency, heteroscedasticity, and normality. [file Data_Sheet_3.pdf]
