# Supplementary material for: Same Invasion, Different Routes: Helminth Assemblages May Favor the Invasion Success of the House Mouse in Senegal
Source: Front Vet Sci. 2021 Oct 26;8:740617. doi: 10.3389/fvets.2021.740617 (PMC8576305; doi:10.3389/fvets.2021.740617)
Supplement: Supplementary Material 4 — Outputs from Chi-squared tests evaluating the difference between host sample sizes in each rodent population. Mus, Mus musculus domesticus; Mastomys, Mastomys erythroleucus; df, degrees of freedom; Ferlo, Central Ferlo; NR3, National Road 3; RV, River valley. Numbers in brackets indicate the sample size. Only sites for which statistical comparisons could be made (i.e., both rodent populations with sufficient number of captured individuals) between M. m. domesticus and M. erythroleucus populations are displayed. The other sites were mostly or exclusively dominated by one or the other rodent species (see Table 1). [file Data_Sheet_4.pdf]

Supplementary Material 4. Outputs from Chi-squared tests evaluating the difference between host sample sizes in each rodent population. Mus: *Mus musculus domesticus*; Mastomys: *Mastomys erythroleucus*; df: degrees of freedom; Ferlo: Central Ferlo; NR3: National Road 3; RV: River valley. Numbers in brackets indicate the sample size. Only sites for which statistical comparisons could be made (i.e., both rodent populations with sufficient number of captured individuals) between *M. m. domesticus* and *M. erythroleucus* populations are displayed. The other sites were mostly or exclusively dominated by one or the other rodent species (see Table 1).

**Sites**	**Relative abundance**	**Chi-squared**	**df**	**p-value**
**Aere Lao (RV)**	Mus (41) > Mastomys (15)	12.071	1	0.000512
**Diomandou Diery (RV)**	Mus (21) = Mastomys (19)	0.1	1	0.7518
**Diomandou Walo (RV)**	Mus (9) < Mastomys (45)	36.255	1	1.73E-09
**Dodel (RV)**	Mus (45) > Mastomys (7)	27.769	1	1.37E-07
**Labgar (Ferlo)**	Mus (26) > Mastomys (6)	12.5	1	0.000407
**Mboumba (RV)**	Mus (6) < Mastomys (48)	32.667	1	1.09E-08
