# Supplementary material for: Same Invasion, Different Routes: Helminth Assemblages May Favor the Invasion Success of the House Mouse in Senegal
Source: Front Vet Sci. 2021 Oct 26;8:740617. doi: 10.3389/fvets.2021.740617 (PMC8576305; doi:10.3389/fvets.2021.740617)
Supplement: Supplementary Material 5 — Outputs from Chi-squared tests evaluating the sex-ratio for each rodent population. “balanced” indicates no statistical gender difference in the number of captured individuals within the rodent population; “biased (females)” indicates that the sex-ratio is biased toward female individuals. Results are significant when p-value < 0.05. df, degrees of freedom. [file Table_2.pdf]

**Supplementary Table 2**. Outputs from Chi-squared tests evaluating the sex-ratio for each rodent population. 'balanced' indicates no statistical gender difference in the number of captured individuals within the rodent population; 'biased (females)' indicates that the sex-ratio is biased towards female individuals. Results are significant when *p-value* < 0.05. df: degrees of freedom.

| | | *Mus musculus domesticus* | | | | *Mastomys erythroleucus* | | | |
|---|---|---|---|---|---|---|---|---|---|
| Zone | Sites | Chi-squared | df | *p-value* | sex-ratio | Chi-squared | df | *p-value* | sex-ratio |
| **River valley** | Aere Lao | 8.3333 | 1 | p-value = 0.003892 | biased (females) | 1.6667 | 1 | p-value = 0.1967 | balanced |
| | Diomandou Diery | 2.3333 | 1 | p-value = 0.1266 | balanced | 0.052632 | 1 | p-value = 0.8185 | balanced |
| | Diomandou Walo | 0 | 1 | p-value = 1 | balanced | 0.021277 | 1 | p-value = 0.884 | balanced |
| | Dodel | 8.0222 | 1 | p-value = 0.004621 | biased (females) | - | - | - | - |
| | Gollere | - | - | - | - | 0.057143 | 1 | p-value = 0.8111 | balanced |
| | Mboumba | - | - | - | - | 2.0833 | 1 | p-value = 0.1489 | balanced |
| | **All sites** | 10.47 | 1 | p-value = 0.001213 | biased (females) | 0.07767 | 1 | p-value = 0.7805 | balanced |
| **Central Ferlo** | Labgar | 0.61538 | 1 | p-value = 0.4328 | balanced | - | - | - | - |
| | Tessekere | 1.6364 | 1 | p-value = 0.2008 | balanced | - | - | - | - |
| | Widou Thiengoly | 1.8148 | 1 | p-value = 0.1779 | balanced | - | - | - | - |
| | **All sites** | 0.33333 | 1 | p-value = 0.5637 | balanced | - | - | - | - |
| **National Road 3** | Dendoudi | 6.7209 | 1 | p-value = 0.009529 | biased (females) | - | - | - | - |
| | Diagali | - | - | - | - | 1.6667 | 1 | p-value = 0.1967 | balanced |
| | Fourdou | - | - | - | - | 2.2727 | 1 | p-value = 0.1317 | balanced |
| | Lambago | 0.25714 | 1 | p-value = 0.6121 | balanced | - | - | - | - |
| | Ranerou | - | - | - | - | 0.037037 | 1 | p-value = 0.8474 | balanced |
| | Yonofere | 0.3913 | 1 | p-value = 0.5316 | balanced | - | - | - | - |
| | **All sites** | 2.8058 | 1 | p-value = 0.09392 | balanced | 1.6667 | 1 | p-value = 0.1967 | balanced |
| All zones | | 2.6964 | 2 | p-value = 0.2597 | balanced | 0.73656 | 1 | p-value = 0.3908 | balanced |
